# Full‐Solution Processed Halide Perovskite Photoanodes with Carbon/NiFe‐LDH Protection for Efficient Photoelectrochemical Water Oxidation

**DOI:** 10.1002/smll.202412713

**Published:** 2025-06-12

**Authors:** Carlos A. Velásquez, Juan J. Patiño, Kevin Ballestas, Franklin Jaramillo, Juan F. Montoya, Daniel Ramírez

**Affiliations:** ^1^ Centro de Investigación Innovación y Desarrollo de Materiales (CIDEMAT) Departamento de Ingeniería de Materiales Facultad de Ingeniería Universidad de Antioquia UdeA Calle 70 No. 52‐21 Medellín 050010 Colombia; ^2^ Grupo de Catalizadores y Adsorbentes (CATALAD) Instituto de Química Facultad de Ciencias Exactas y Naturales Universidad de Antioquia UdeA Calle 70 No. 52‐21 Medellín 050010 Colombia

**Keywords:** carbon‐based photoelectrodes, green hydrogen, hybrid perovskites, NiFe‐LDH catalyst, water oxidation

## Abstract

The development of photoelectrochemical water oxidation (PEC) systems has gained significant relevance in recent years due to the quest for clean fuels, where green hydrogen is one of the main actors in the energy transition. Particularly, there has been a need for precious metal‐free electrodes and photoelectrodes that demonstrate high efficiency and stability aiming at having large‐scale and low‐cost green hydrogen production systems. This work shows advances toward this goal by using nonprecious catalysts and solution‐processed materials to achieve efficient, low‐cost, and stable photoelectrodes. Specifically, carbon‐based hybrid perovskite photoelectrodes coupled with an earth‐abundant Nickel‐Iron layered double hydroxide (NiFe‐LDH) catalyst (carbon/NiFe‐LDH) are fabricated and evaluated for oxygen evolution reaction (OER). Devices with an active area of 1.1 cm^2^ exhibit evaluated over 12 h of continuous operation, a 4.57% ABPE at 0.64 V_RHE_, and a photocurrent density of 11.71 mA cm^−2^ at 1.23 V_RHE_. The incorporation of graphite tape results in a system (C/GT/NiFe‐LDH) that shows an exceptional operational stability over 125 h and efficiency for this type of photoelectrode with a high photocurrent density of 18.07 mA cm^−2^ at 1.23 V_RHE_ and 8.51% ABPE at 0.67 V_RHE_.

## Graphical abstract

Solution‐processed photoelectrodes for the oxygen evolution reaction in water splitting are fabricated by using hybrid perovskite as the active layer, carbon electrodes, and NiFe‐LDH catalyst as protective and catalyst layers, respectively. The device showed an 18.07 mA cm^−2^ photocurrent density and an 8.51% ABPE.

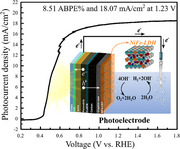

## 1 Introduction

Due to growing concerns with respect to climate change, renewable energy sources have become of great importance, consequently leading to an increase in research and development on the topic driven by global policies aiming to reduce the carbon footprint of energy sources.^[^ ^1^, ^2^ ^]^ In this context, solar cells have emerged as a cornerstone in the transition to cleaner energy sources.^[^ ^3^, ^4^, ^5^, ^6^, ^7^ ^]^ Recently, perovskite‐based third‐generation solar cells have gained attention for their potential low cost, ease of processing, and flexibility, offering new opportunities in photovoltaic applications.^[^ ^4^, ^8^, ^9^ ^]^ The applications of this type of material goes beyond direct photovoltaics, showing promise in the production of green hydrogen through water splitting when integrated with low‐cost catalysts and carbon‐based protective materials to create efficient photoelectrodes (photocathodes and/or photoanodes).^[^ ^10^, ^11^, ^12^, ^13^, ^14^, ^15^, ^16^, ^17^ ^]^ In these devices, the carbon‐based protective materials, which usually also serve as electrodes, are typically processed using mixtures partially consisting of a polymeric matrix or various liquid phase solvents that must be compatible with the perovskite material in order to avoid its degradation during fabrication.^[^ ^18^ ^]^ Solid carbonaceous materials are key for obtaining a suitable conductivity.^[^ ^19^, ^20^ ^]^ The combination of appropriate polymer matrix and carbon materials enhances the hydrophobicity and compactness of the electrode, allowing water to be repelled from the surface and preventing infiltration of the aqueous medium (where the water splitting reaction takes places) inside the device.^[^ ^10^, ^21^, ^22^, ^23^ ^]^ In addition to this carbon‐based protective layer, a flexible graphite sheet can also be deposited on top of this electrode to get better protection.^[^ ^12^, ^13^, ^21^, ^24^, ^25^, ^26^, ^27^ ^]^

Currently, PEC devices based on third‐generation perovskite solar cells have achieved high efficiency for the water splitting reaction with high stability times.^[^ ^22^, ^24^, ^28^, ^29^ ^]^ Considering that in such devices the perovskite materials are usually in direct or nearly direct contact with the electrolyte medium, a challenge arises for this technology due to the intrinsic instability of halide perovskites to water. To overcome this, several strategies have been reported in the literature, such as the use of nonaqueous solvents as reaction media, the use of compact tapes or ultrathin metal layers deposited by atomic layer deposition (ALD) for increasing device stability, as well as the use of carbon electrodes.^[^ ^10^, ^22^, ^24^, ^30^, ^31^, ^32^, ^33^, ^34^ ^]^ As a result, high values of photocurrent density (*J*_sc_) and low onset potentials have been reported in literature, showing ranges of 0.4–22 mA cm^−2^ and 0.35–0.80 V_RHE_, respectively (see Table S1, Supporting Information). Thus, these systems have been positioned as a viable alternative to those commonly used for water splitting, which are based on other materials, namely TiO_2_, BiVO_4_ and Fe_2_O_3_, among others.^[^ ^35^, ^36^, ^37^, ^38^, ^39^ ^]^ An approach to protect the perovskite material on these devices is to deposit a protective film on top of it, which in turn is usually coupled with a catalytic material. In this context, carbon‐based materials coupled with suitable catalysts have been demonstrated to work reasonably well both by themselves as electrodes and integrated into photoelectrodes,^[^ ^40^ ^]^ reaching values of solar‐to‐hydrogen efficiency (STH) as high as 20.8%.^[^ ^12^ ^]^

Catalyst materials, crucial for optimizing the performance of photoelectrodes, have been extensively studied for their ability to reduce the energy required for reactions or alter reaction mechanisms.^[^ ^40^, ^41^, ^42^, ^43^ ^]^ Transition metal oxides and hydroxides from elements such as Mn, Fe, Co, and Ni are particularly interesting in this context due to their low cost, abundance, and high catalytic activity.^[^ ^14^, ^44^, ^45^ ^]^ When used under high alkalinity conditions, catalysts with a layered double hydroxide (LDH) structure stand out thanks to the low overpotentials (η) that can be attained. This η represents the additional energy required to surpass the thermodynamic barrier needed for the reaction to occur, which ideally is 1.23 V with respect to the Normal Hydrogen Electrode (NHE) in the case of water splitting. Incorporating Fe into the catalyst structure coupled with Ni could significantly enhance the oxygen evolution reaction (OER) due to their synergistic effect, leading to improved adsorption on the catalyst surface and faster charge transfer. This type of NiFe‐LDH catalytic material has demonstrated reductions of η in water splitting reactions, resulting in savings in both costs and required processing time.^[^ ^41^, ^46^ ^]^ There are several methods for synthesizing such type of material, as well as a wide variety of deposition techniques. Most of these catalysts are deposited onto a substrate, and the most cost‐effective methods for this are solution‐based, e.g., dip‐coating, spray pyrolysis, and other spray coating deposition methods, which can achieve suitable morphologies and high surface areas, thereby enhancing the catalytic efficiency.^[^ ^47^, ^48^ ^]^

For the practical application of the PEC in water splitting it is essential to obtain suitable PEC parameters, adequate stability in aqueous electrolytes, and scale up the photoelectrode, which is directly related to the ease of processing the multilayers that comprise a large evaluation area without hindering its performance. To meet all the above requirements, in this work, a full‐solution processed photoanode was fabricated using a perovskite solar cell with a carbon‐based protective electrode coupled with a NiFe‐LDH catalyst developed in previous studies.^[^ ^40^ ^]^ Initially, the deposition of the hole transporting layer (HTL) of the device, in this case CuSCN, was carried out by evaluating different deposition cycles on the same film from a solution at the maximum possible concentration, which led to the formation of multilayers rather than the removal of previously deposited layers. A methodology for the fabrication of the carbon electrode was developed to obtain the best photovoltaic parameters for carbon‐based solar cells. Then, photoanodes were produced with the subsequent deposition of the NiFe‐LDH catalytic material to promote the oxygen evolution reaction (OER), achieving stability for 12 h in contact with an aqueous solution under continuous solar irradiation. It is highlighted that thicknesses of 40–60 and 1.5 µm were achieved for the carbon electrode and the NiFe‐LDH catalyst, respectively, with the carbon electrode reaching a sheet resistance of 25 Ω sq^−1^. Additionally, the incorporation of a conductive graphite tape improved the performance of the photoanode with a high performance over 125 h. This enabled the development of a PEC device with a combination of protective and catalytic layers that can be processed in solution using scalable and low‐cost methods to carry out the OER with high efficiency and stability over a large active area of 1.1 cm^2^.

## 2 Results and Discussion

### 2.1 Fabrication and Device Performance of Full Solution‐Processed Carbon‐Based Perovskite Solar Cells (C‐PSCs)

**Figure 1:**
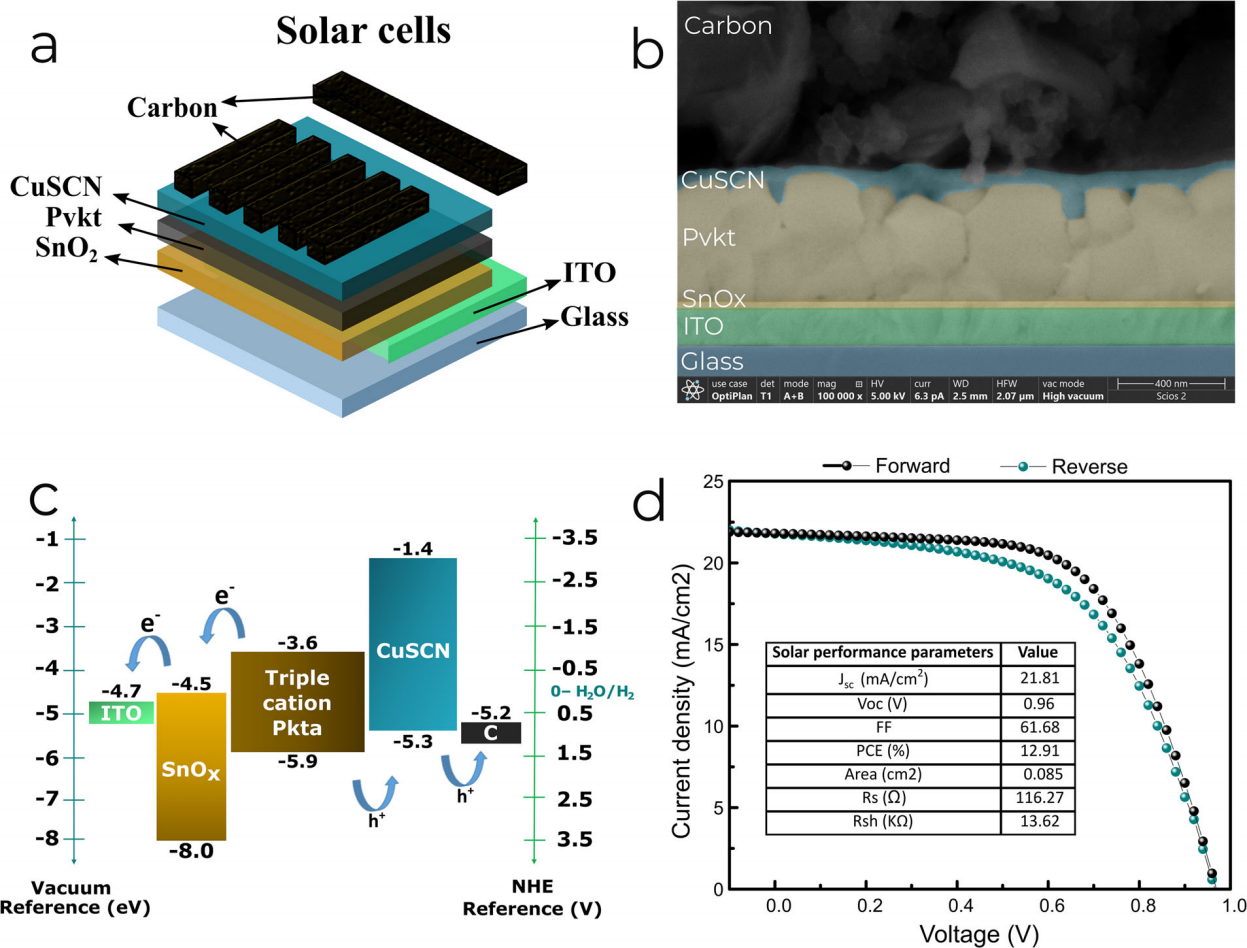
a). Schematic carbon‐based perovskite solar cell structure, b) cross‐section SEM image of C‐PSC fabricated using 2 deposition cycles of CuSCN, c) band diagram of the structure used and d) *J–V* curve of the best‐performing C‐PSC with photovoltaic parameters.

Full solution‐processed n‐i‐p solar cells with carbon electrode were fabricated and optimized for the structure: glass/ITO/SnO_x_/Cs_0.05_(FA_0.9_MA_0.1_)_0.95_Pb(I_0.9_Br_0.1_)_3_/CuSCN/carbon to later be used as the base for the subsequent fabrication of the photoelectrodes. CuSCN was selected as HTL due to its solvent compatibility with the underlying perovskite, nonsolubility in water, thus offering good protection against moisture, high thermal stability, and high hole mobility.^[^ ^49^ ^]^ Additionally, it is a cheap and abundant p‐type semiconductor that has been demonstrated to form a stable interface with the underlying perovskite that does not undergo reactions under illumination. Instead, a possible electrical potential‐induced reaction with gold is regarded as a degradation mechanism when employed as an HTL, which would not be a concern here since it is coupled with carbon.^[^ ^49^, ^50^ ^]^ The optimization process consisted first of the morphological and structural evaluation of a triple cation perovskite layer to determine the appropriate phases and morphology (see Figure S1, Supporting Information).^[^ ^51^ ^]^ X‐ray diffraction (XRD) showed the presence of typical α‐phase triple cation perovskite peaks at 2*θ* values of 14.34°, 20.4°, 24.84°, 28.7°, 32.2°, 35.3°, 40.1°, and 43.6°. Two additional peaks at 11.6° and 12.7 ° are also present and correspond δ‐phase FAPbI_3_ and PbI_2_ in the cubic phase, respectively (see Figure S1a, Supporting Information).^[^ ^51^, ^52^ ^]^ All peaks are commonly found in triple cation perovskite films and overall indicate an adequate phase purity for photovoltaic operation. Then, the optimization of the CuSCN HTL layer was carried out by varying the thicknesses through spin coating processing, in which the number of applied coatings was varied to find the best morphological and optical condition (see Figures S2 and S3, Supporting Information). Finally, a carbon electrode was implemented as the final layer, replacing the commonly used metallic materials in third‐generation solar cells, to achieve optimal electrical and protective properties discussed throughout the work as well as adequate photovoltaic parameters for devices with 2 depositions (cycles) of CuSCN (see Figure S4, Supporting Information). The schematic configuration of the carbon‐based perovskite solar cells (C‐PSCs) is shown in **Figure** **1**a, each device consisting of 5 sub‐cells. The carbon electrode was developed with suitable electrical properties, such as a sheet resistance of 25 Ω sq^−1^ and thicknesses of 40–60 µm. These requiree charge carriers to travel a long distance to reach the external circuit. However, the development of the carbon electrode was not solely focused on adequate electrical properties; it also aimed to achieve sufficient thickness and compactness to protect the devices.  

A cross‐section scanning electron microscopy (SEM) image of the fabricated C‐PSC is shown in Figure 1b, where it is possible to identify the thickness and the interfaces between layers. At the CuSCN/carbon interface, some points of contact through the nanoparticles that make up the carbon layer can be observed, which allow for the extraction of photogenerated holes. It is also evident that 2 cycles of CuSCN deposition created a compact layer rather than just a coating on the perovskite grains. Figure 1c shows the band diagram of the stack, from which it can be inferred that the alignment between the carbon work function and the valence band of CuSCN allows hole transport.^[^ ^53^, ^54^, ^55^, ^56^ ^]^ This is the main reason why solar cells exhibited a good voltage, considering that the typical band gap for triple cation perovskites ranges from 1.1 to 2.3 eV, but the specific composition used in this work is within the range of 1.5–1.6 eV.^[^ ^51^ ^]^

In Figure 1d, the *J–V* curve of the best‐performing C‐PSC displays a J_SC_ 22 mA cm^−2^, a remarkable value for a solar cell with a carbon electrode, considering that charge transport is limited by a much greater thickness and lower conductivity compared to metallic electrodes, namely Ag and Au. Also, a *V*_OC_ of 0.96 V was achieved, which is attributed to proper band alignment with the work function of the carbon electrode (see Figure 1c), determined in previous works to be ≈5.24 eV.^[^ ^40^, ^57^ ^]^ The limiting parameter for photovoltaic performance in this case is the fill factor (FF), which depends on the quality of the interfaces. However, the efficiency of the C‐PSC obtained is comparable to the state of the art.^[^ ^58^ ^]^ In fact, one notable aspect of this work is the achievement of a solar cell with a 12.91% efficiency using a solution processed carbon electrode, along with a solution processed inorganic CuSCN layer.

### 2.2 Photoanodes for the Oxygen Evolution Reaction in Water Splitting

#### 2.2.1 Photovoltaic Characterization

To develop the PEC system, devices with a larger active area (1.1 cm^2^) were fabricated. In addition to sole carbon electrodes, carbon (C) /graphite tape (GT) electrodes were also tested, since GT potentially enhances water resistance, given a suitable electrical contact layer couples it with the device, in this case the carbon layer.^[^ ^10^ ^]^ Note that current density and voltage are the most relevant parameters for the subsequent electrochemical evaluation. Therefore, solar cells that achieved the best photovoltaic parameters were selected for the fabrication of the large‐area photoelectrodes. Commonly, large‐area solar cells show lower power conversion efficiency (PCE) than their smaller area counterparts, which can be caused by interface defects, contamination, or defects inherent to the film, such as differences in thickness or pinholes, which have a higher probability of occurrence on larger areas.^[^ ^59^, ^60^ ^]^ These factors account for the lower photovoltaic parameters achieved in comparison to those reported for small‐area devices (Figure 1).

**Figure 2:**
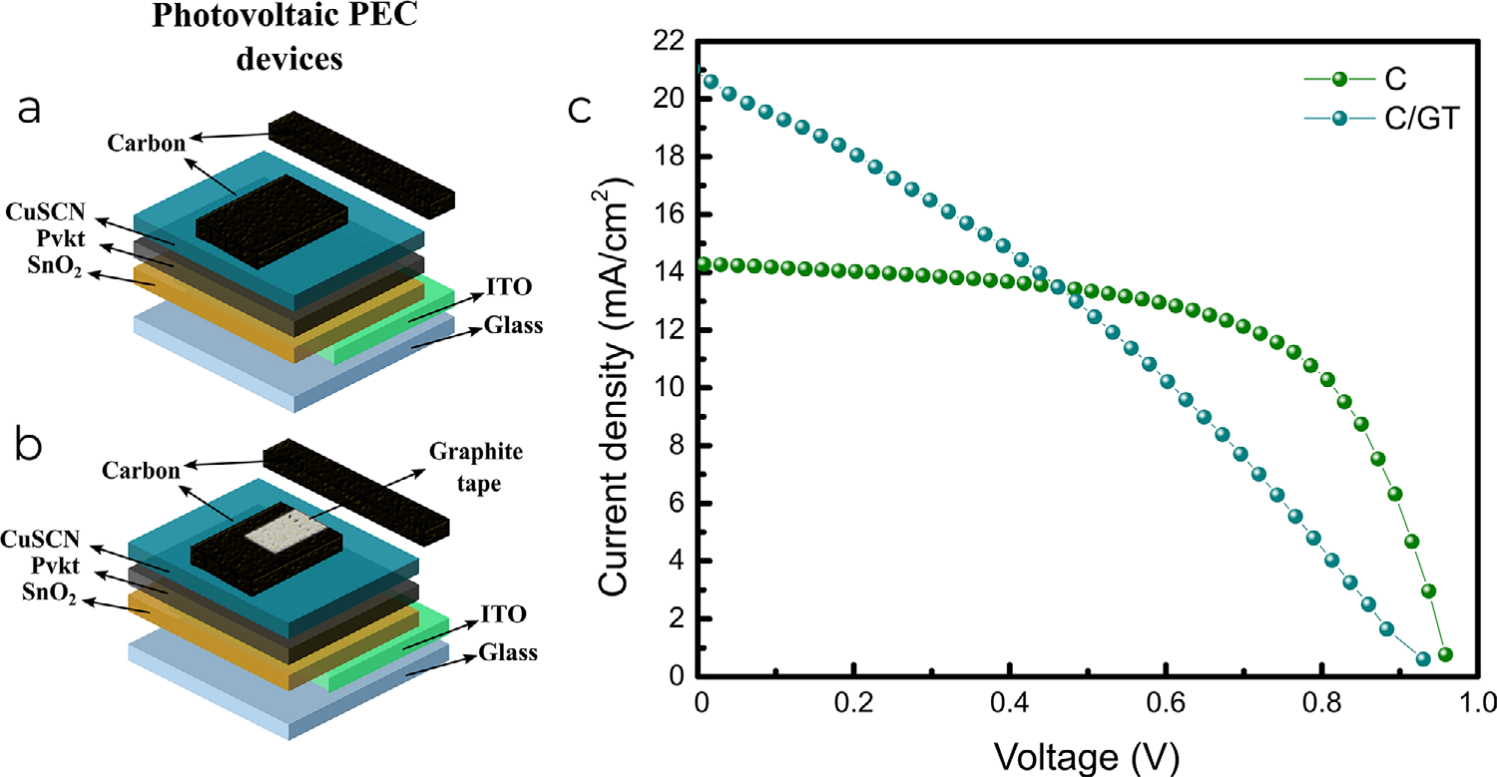
Large area C‐PSCs used for subsequent evaluation as photoelectrode. Schematic structure of a) devices with carbon and b) C/GT electrodes. c) *J–V* curves of best‐performing large area devices with carbon and C/GT electrodes.

**Figure** **2**a,b displays the schematic devices with large active area featuring carbon and C/GT electrodes, respectively. The achieved *V*_OC_ and *J*_SC_ values indicated that the C‐PSC could be utilized in a PEC device for water splitting, incorporating the catalyst material as a top layer through a solution processing method. Figure 2c shows that the *J*_SC_ obtained for the best‐performing devices was ≈21 mA cm^−2^ for the configuration with GT and ≈14–15 mA cm^−2^ for the configuration without it. The lower values of the latter may be linked to the quality of the films and interfaces developed during processing. This parameter limits the photocurrent density achievable in PEC devices, as they behave similarly to a diode. The maximum J_SC_ obtained for the solar cell will not be exceeded when used in a PEC device. On the other hand, the *V*_OC_ achieved by best‐performing C‐PSCs is close to 1 V, which is the maximum voltage that the solar cell can provide to the PEC devices. This translates to a direct energetic contribution from the C‐PSC to the water splitting reaction, which is clearly observed in linear sweep voltammetry (LSV) as an onset of the reaction below the theoretical 1.23 V, as discussed later. It is noteworthy that the photovoltaic parameter that has the largest room for improvement in large‐area cells is the FF. This has been previously reported to be related to the higher probability of finding defects in larger areas.^[^ ^61^, ^62^ ^]^

#### 2.2.2 Morphological Characterization

**Figure 3:**
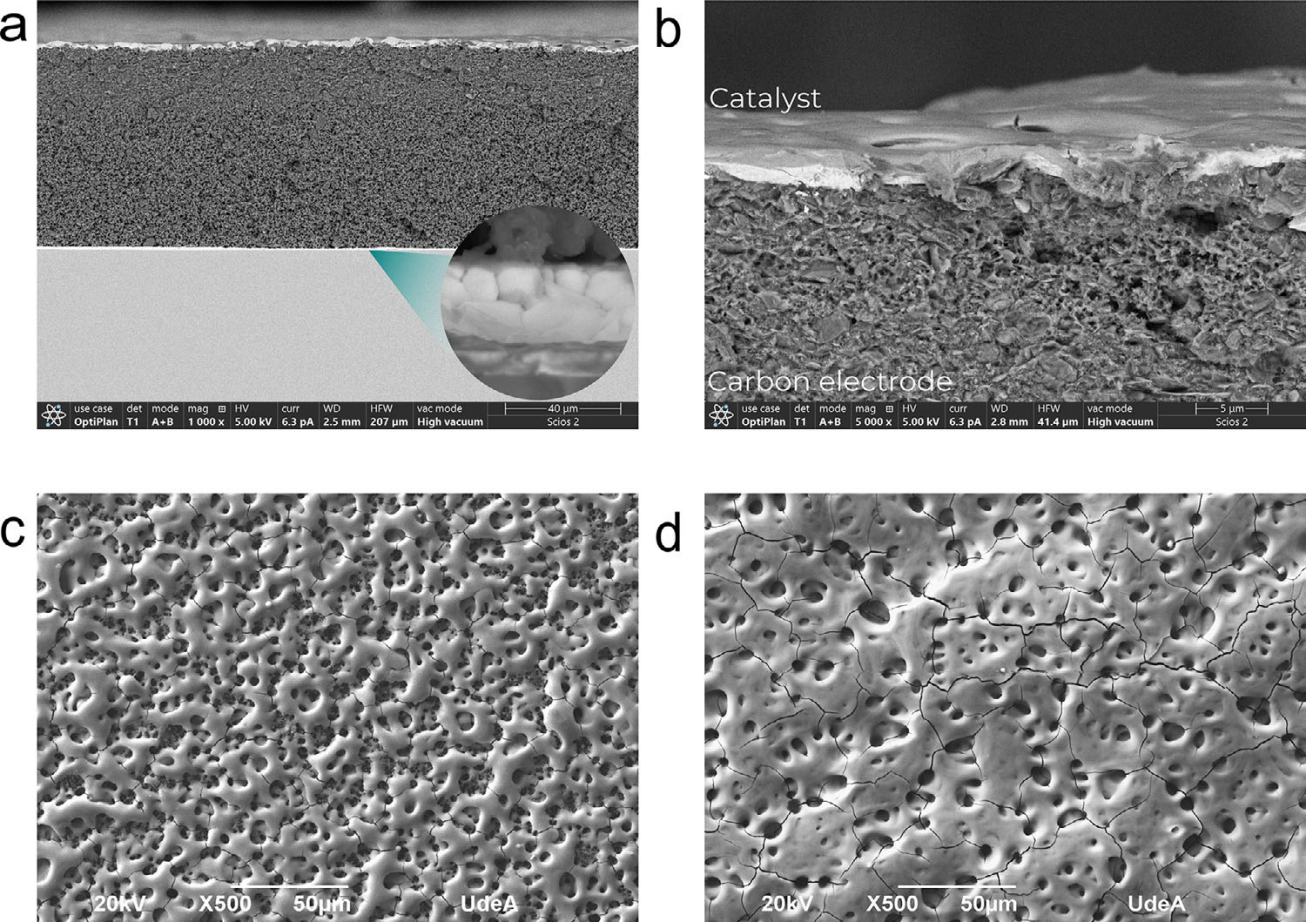
SEM images of PEC device fabricated with C‐PSC. a) Cross‐section of the multilayer PEC device at 1000X with an inset image showing the interfaces of the perovskite layer at a magnification of 100.000X. b) C/NiFe‐LDH interface at 5000X and top view at 500X of c) C/NiFe‐LDH and d) C/GT/NiFe‐LDH.

The glass/ITO/SnO_x_/Cs_0.05_(FA_0.9_MA_0.1_)_0.95_Pb(I_0.9_Br_0.1_)_3_/CuSCN/C/NiFe‐LDH system was morphologically characterized to determine the coupling between of each layer by using images of field emission scanning electron microscopy (FESEM) for the cross‐sections shown in **Figure** **3**a,b, as well as the top view for the catalyst deposited on top of the carbon electrode (Figure 3c) and C/GT (Figure 3d).

Figure 3a shows the cross‐section of the fabricated photoanode by coupling C/NiFe‐LDH electrode. The carbon electrode has visible features an intimate contact between the components of the carbon paste. Some micro‐ and nano‐pores are also present, indicating that the electrode is not uniformly compact across its 40–60 µm thickness. However, this structure promotes a tortuous path that could prevent electrolyte infiltration into the underlying layers and consequently avoids moisture contact with the perovskite, leading to increased stability over time. Additionally, Figure 3a,b further reveals a good interaction between the carbon and NiFe‐LDH layers, with no significant gaps between them, suggesting a stable catalyst/electrode interface. Figure S5 (Supporting Information) presents in‐situ Raman evidence supporting the formation of a compact NiFe‐LDH structure and a stable interface with the carbon substrate during the device operation. The persistence of the CO₃^2^⁻ band, despite its reduced intensity at 0.6 V, indicates limited ion exchange and a dense interlayer, which likely hinders electrolyte or moisture infiltration, contributing to enhanced structural stability. Additionally, the decrease in the I_D_/I_G_ ratio at 0.2 V suggests improved carbon ordering and stronger interfacial coupling with the NiFe‐LDH layer. At 0.6 V, the suppression of the D and G bands is attributed to the formation of a NiOOH/FeOOH overlayer, which spectroscopically shields the carbon, confirming complete catalyst coverage and intimate contact with the substrate. Furthermore, Figure S5 (Supporting Information) provides detailed insights into the chemical transformations occurring during the OER, highlighting their influence on the catalyst's structural evolution and electrochemical activity. Complementary morphological analysis (Figure 3c,d) reveals a continuous catalyst layer ranging from 1.5 to 2 µm in thickness, with full surface coverage over both carbon and C/GT electrodes. The observed porous morphology, characterized by numerous cavities, increases the active surface area and enhances the catalytic performance during water splitting.^[^ ^41^ ^]^ Notably, the cavity walls are thinner in the C/NiFe‐LDH system compared to the C/GT/NiFe‐LDH system, a difference attributed to the higher surface roughness of the carbon electrode. This contrasts with the smoother GT substrate, which is composed of compact layered sheets. Additionally, the GT exhibits greater wettability (Figure S6, Supporting Information), influencing the catalyst‐substrate interaction during spray coating. Differences in heat dissipation between the systems, higher in C/NiFe‐LDH than in C/GT/NiFe‐LDH, also affect catalyst deposition, as heat must transfer through the carbon before reaching the GT in the latter, impacting droplet behavior and film formation.^[^ ^40^, ^41^ ^]^

### 2.3 Oxygen Evolution Reaction During Water Splitting

Electrochemical characterization was performed by means of a PEC reactor while subjecting the photoelectrode to 1 sun illumination in a three‐electrode configuration (see Figures S7 and S8, Supporting Information), and the applied bias photon‐to‐current efficiency (ABPE) was calculated using Equations (2 and 3) from the experimental section and experimental data obtained from linear sweep voltammetry (LSV).

**Figure 4:**
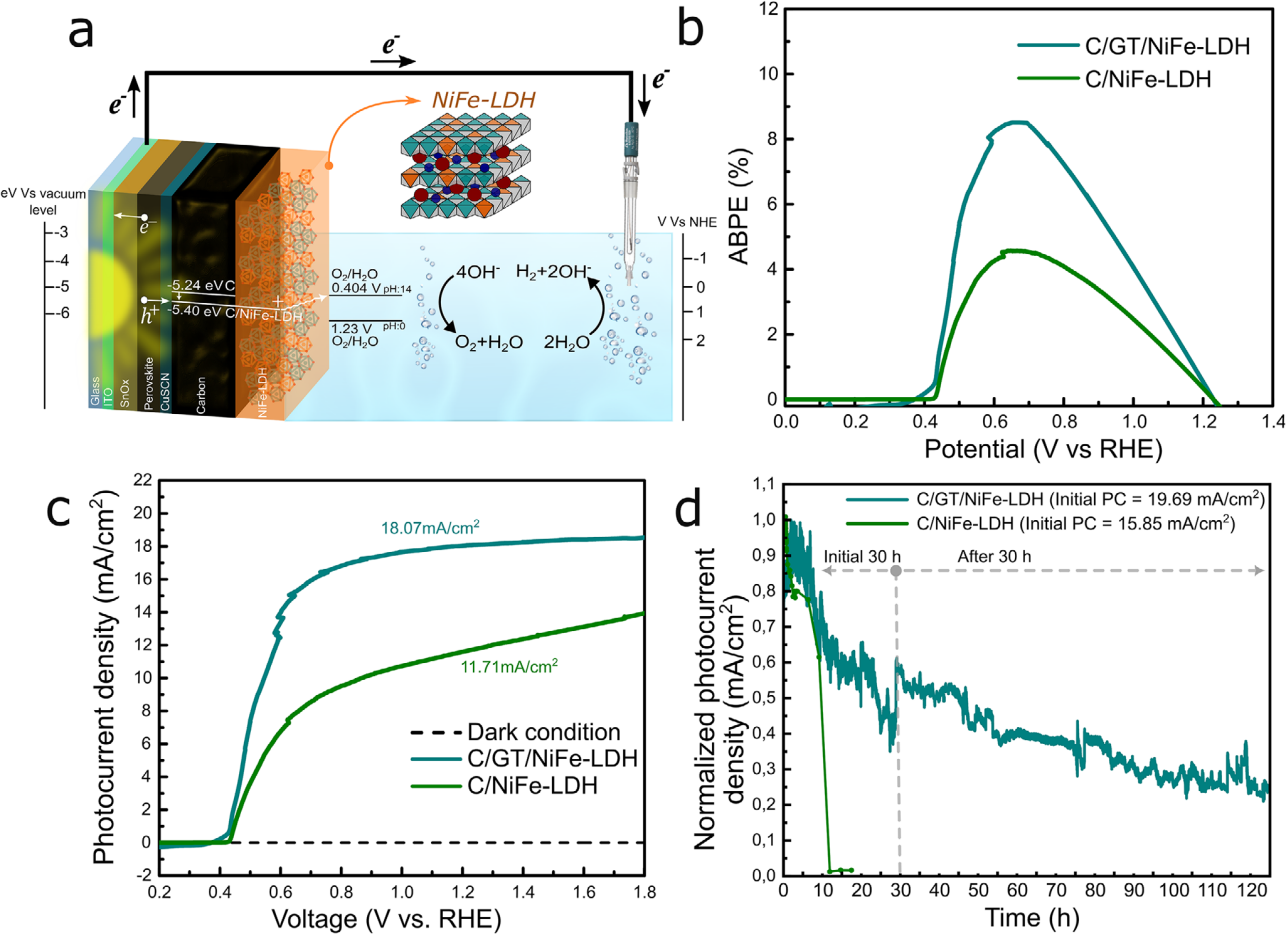
PEC characterization of the devices ITO/SnO_x_/ Cs_0.05_(FA_0.9_MA_0.1_)_0.95_Pb(I_0.9_Br_0.1_)_3_/CuSCN/C/NiFe‐LDH and ITO/SnO_x_/ Cs_0.05_(FA_0.9_MA_0.1_)_0.95_Pb(I_0.9_Br_0.1_)_3_/CuSCN/C/GT/NiFe‐LDH. a) Schematic diagram of photoanode with C/NiFe‐LDH as an OER catalyst and energy level diagram of C/ NiFe‐LDH. b) APBE (%) as a function of applied bias. c) LSV and d) chronoamperometry of photoanodes evaluated for 12 h (C/NiFe‐LDH) and 125 h (C/GT/NiFe‐LDH)

**Figure** **4**a shows the scheme of the photoelectrode developed from the n‐i‐p C‐PSC illustrated in Figure 2a,b. This configuration showed suitable photovoltaic performance for the subsequent incorporation of the NiFe‐LDH catalyst, with the latter acting as the material in direct contact with the electrolyte through which the OER reaction will take place.

Therefore, Figure 4a shows the work function levels of both the carbon electrode and the NiFe‐LDH catalyst. This was determined using the Kelvin probe force microscopy (KPFM) method via atomic force microscopy (AFM, experimental details in Ref.[40]). From this, an adequate energy level matching for the interfacial charge transfer process from the catalytic material to the electrolyte is observed. In a basic electrolyte medium, such as the one used in this study, a shift in the redox potential to a lower value (V vs RHE) occurs, which facilitates the oxygen evolution reaction (OER) by providing a more efficient charge transfer pathway at the C/electrolyte interface. Furthermore, the coupling of the catalytic material with the carbon (C/NiFe‐LDH) leads to a shift in the work function (eV vs vacuum level) toward more negative values, further enhancing the charge transfer from the catalyst to the electrolyte. This energy level alignment reduces the required overpotential and improves the overall performance of the system as detailed more thoroughly in a previously published study.^[^ ^40^ ^]^

On the other hand, Figure 4b shows the ABPE achieved by the PEC devices, noting that the system under evaluation is supplied with additional energy through a potentiostat. ABPE is defined as the ratio of the incident solar energy converted into hydrogen energy under a specific applied bias, resulting in a graph of ABPE versus voltage (*V*_RHE_, voltage with respect to the reversible hydrogen electrode). From the analysis of Equation (3), it can be inferred that the ABPE will depend on the photocurrent density (*J*_ph_) achieved by the devices, as well as the starting potential (*V*_on_) of the LSV. In fact, *V*_on_ parameter is inversely and *J*_ph_ is directly proportional to ABPE, and thus it is essential to seek high initial photovoltaic parameters to attain greater efficiency in hydrogen production with PEC devices with a large active area of 1.1 cm^2^.^[^ ^24^ ^]^ The photoelectrodes with C/NiFe‐LDH showed an ABPE of 4.57% at 0.64 *V*_RHE_, while the configuration with C/GT/NiFe‐LDH showed an exceptional ABPE of 8.51% at 0.67 *V*_RHE_.

In Figure 4c, a comparison is made between the devices with the C/NiFe‐LDH and C/GT/NiFe‐LDH configurations. It is evident that the system with GT achieves a high *J*_ph_ of 18.07 mA cm^−2^ at 1.23 *V*_RHE_ compared to the structure without it, which achieves a *J*_ph_ of 11.71 mA cm^−2^ at 1.23 *V*_RHE_. This can be attributed to the conductivity provided by the GT when coupled with the catalyst, allowing for more charges to interact with the electrolyte. Nonetheless, the *J*_ph_ values obtained for the C/NiFe‐LDH system are promising for hydrogen generation applications due to the abundance of the materials involved and their ease of processing. In Figure 4c, it is also possible to observe that both configurations achieved a similar onset potential (*V*_on_) of 0.37 and 0.40 *V*_RHE_ for C/GT/NiFe‐LDH and C/NiFe‐LDH, respectively, which aligns well with the photovoltaic performance shown in Figure 2c. The V_OC_ achieved by the solar cell itself should be fully reflected in the *V*_on_, implying that in an ideal system, the *V*_on_ of the PEC devices should start at ≈0.3 V. However, in these types of systems, potential losses generated during the process must be considered,^[^ ^63^ ^]^ as shown in Equation (1), where 1.23 V corresponds to the thermodynamic potential to carry out the OER reaction under standard conditions of 25 °C, 1M_(aq),_ and 1 atm, *V*_pa_ (V) is the operating photovoltage of the photovoltaic cell which is converted to photoanode, and η_OER_ (V) is the overpotential for OER catalyst. When coupling the catalyst as an additional layer, it may alter the transport and charge transfer processes from the device to the electrolytic medium, leading to a decrease in *V*_on_.

(1) $$V_{\mathrm{photoanode}}=1.23V-V_{pa}+\eta_{OER}$$

The low values of *V*_on_ and the increase in *J*_ph_ in the potential range of 0.45–6 V_RHE_ lead to high ABPE values associated with two effects that occur during the charge transport process. First, the FF^[^ ^24^ ^]^ achieved by the C‐PSCs influences the transport of generated charges from the semiconductor material to the external system, as evidenced in Figure 2c. It can be observed that the C/NiFe‐LDH system presents an adequate FF, while the C/GT/NiFe‐LDH system does not show an adequate FF, which is associated with the suboptimal mechanical coupling between the GT and the carbon electrode when measured as large‐area solar cells. However, when the system is coupled to the measurement system (see Figure S8, Supporting Information), it is usually sealed through mechanical pressure, leading to greater contact between the GT and the carbon electrode, thereby enhancing the transport of charge carriers from the carbon to the GT, as shown in Figure 4c. Second, the improvement in charge separation efficiency (η_surface_) due to the kinetics of charge transfer across the NiFe‐LDH interface and the electrolyte also plays a role in the enhancement of ABPE.^[^ ^24^, ^35^, ^36^, ^64^ ^]^ It is important to highlight the capability of the sole C/NiFe‐LDH system, as it achieves high *J*_ph_ values at low potentials, allowing it to display a high ABPE compared to other PEC systems that are frequently based on scarce and more expensive materials.^[^ ^22^, ^24^, ^34^ ^]^

For the C/GT/NiFe‐LDH system, Figure 4d shows that the combination of carbon and GT protective layers yields promising device stability, retaining 45% of the initial photocurrent (19.69 mA cm^−2^) after 30 h of operation. Notably, a slight increase in photocurrent is observed during the initial hours, attributed to enhanced charge transport under applied potential, an effect previously reported in perovskite solar cell technologies.^[^ ^65^ ^]^ The chronoamperometric stability test was divided into two phases. The first 30‐h segment was selected to simulate a realistic operational timespan. However, the device retained a substantial portion of its photocurrent beyond this period, prompting a second measurement phase of an additional 95 h. Interestingly, the photocurrent at the start of the second phase exceeded the value at the end of the first 30 h. This behavior is commonly observed in solar devices due to charge accumulation at the interfaces during prolonged illumination. Upon light cycling, the release of these accumulated charges can transiently enhance the photocurrent.^[^ ^66^ ^]^ Following this, the photoanode exhibited a slower degradation rate, maintaining 25% of its initial photocurrent after 125 h of operation. This underscores the effectiveness of the combined GT and carbon layers in enhancing long‐term stability. Additionally, the GT layer acts as a compact protective barrier that delays device degradation, as evidenced by the post‐operation electrode images in Figure S9 (Supporting Information).^[^ ^10^, ^11^ ^]^ Motivated by these findings, we extended the stability evaluation to the C/NiFe‐LDH system alone. In this case, the device exhibited a gradual decline in photocurrent from 15.85 to ≈12 mA cm^−2^ within the first 7 h, followed by a further drop to ≈10 mA cm^−2^ by hour 10. Complete device failure, indicated by zero photocurrent, occurred between 10 and 12 h (Figure S10, Supporting Information). While some devices reported in the literature exhibit longer operational stability, they often rely on compact metallic films or rare, costly metal oxides (see Table S1, Supporting Information). In contrast, the carbon‐based systems explored here, despite shorter lifetimes, offer a low‐cost, scalable, and sustainable alternative with significant potential for further development and optimization.

Multiple devices with C/GT/NiFe‐LDH and C/NiFe‐LDH configurations were fabricated and evaluated as large‐area solar cells, with their corresponding *J–V* curves obtained. Additionally, electrochemical characterization was performed to assess their average performance, with statistical results presented in Figure S11 (Supporting Information). Although several devices were analyzed, the main manuscript focuses on those that exhibited the best photovoltaic parameters, as these also achieved outstanding performance in the oxygen evolution reaction (OER), as discussed throughout the paper.

**Figure 5:**
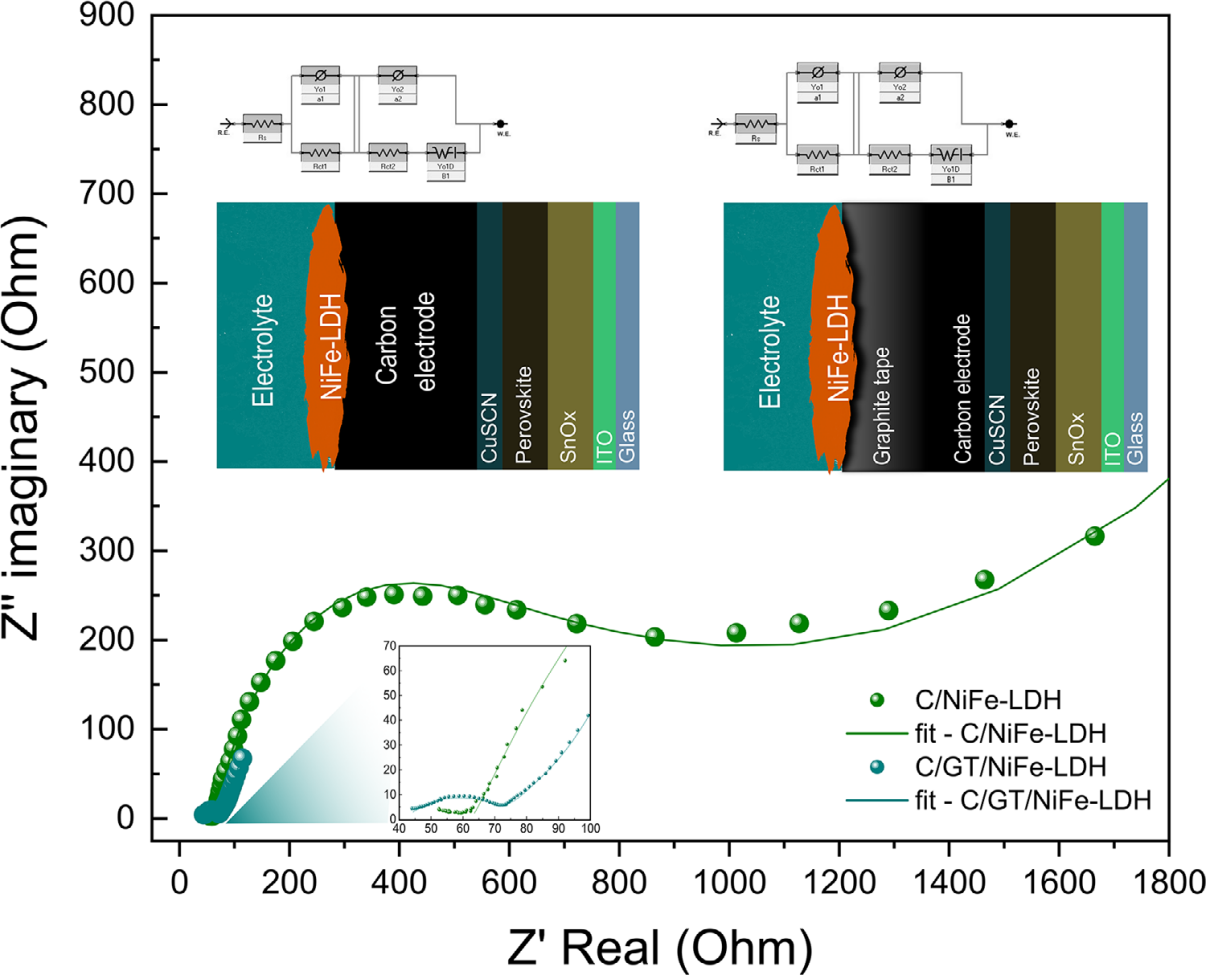
Comparative EIS of photoanodes using C/NiFe‐LDH and C/GT/NiFe‐LDH and the corresponding equivalent circuits.

The electrochemical charge transfer behavior of both systems was evaluated, as shown in **Figure** **5**, where the charge transfer is graphically represented at the endpoint of the semicircle. The electrochemical impedance data indicate that both systems exhibit low charge transfer resistance, which is necessary for an efficient water splitting reaction. However, upon comparison, the C/GT/NiFe‐LDH system demonstrates a lower charge transfer resistance than the C/NiFe‐LDH system. This finding supports the data shown in Figure 4, as lower charge transfer resistance facilitates more efficient current flow from the system to the electrolyte.

The main difference between the C/NiFe‐LDH and C/GT/NiFe‐LDH systems lies in the low resistivity of the GT in conjunction with carbon, which provides greater ease and speed in transporting charges to the catalytic material, as corroborated by electrochemical impedance spectroscopy (EIS) evaluation, where the difference in charge transfer resistance between both interfaces (*R*_ct1_ and *R*_ct2_ as shown in Table S2, Supporting Information) of every systems is evident (see Figure 5; Table S2, Supporting Information). The charge transfer resistance *R*_ct1_ is lower in the C/GT/NiFe‐LDH system compared to the C/NiFe‐LDH system, indicating more efficient charge transfer at the catalyst/electrolyte interface. This difference in *R*_ct1_ values is attributed to the specific structure of the catalyst, as previously discussed. Meanwhile, *R*_ct2_ corresponds to the charge transfer at the GT/catalyst and C/catalyst interfaces. The lower resistance in the GT is due to its higher conductivity compared to carbon, which significantly enhances charge transfer The diffusivity coefficient calculated using Equation S3 and S4 (see Table S2, Supporting Information) for both systems reflects the incorporation of the electrolyte through the pores of the carbon layer, which accounts for the similarity between the values for both configurations. This suggests that over time, the electrolyte can infiltrate the GT/C system, leading to the observed type of interaction.

## 3 Conclusions

Full solution‐processed photoanodes were developed based on the optimal configuration of C‐PSC for the water oxidation reaction. These devices were protected using a carbon electrode and graphite tape, onto which a catalytic material of NiFe‐LDH was deposited via spray coating, denoted as C/NiFe‐LDH and C/GT/NiFe‐LDH, respectively. Under 1 sun illumination, the photocurrent density values, turn‐on voltages, and ABPE for the configuration with carbon/NiFe‐LDH were 11.71 mA cm^−2^ at 1.23 V_RHE_, 0.40 *V*_RHE_, and 4.57% at 0.64 *V*_RHE_, respectively. On the other hand, for the system with C/GT/NiFe‐LDH, these values were 18.07 mA cm^−2^ at 1.23 V_RHE_, 0.37 *V*_RHE_, and 8.5% at 0.65 *V*_RHE_. This is evidence of the great electrical and catalytic capacity of both configurations, especially the C/GT/NiFe‐LDH system, which showed significant kinetics of charge transfer across the NiFe‐LDH interface and the electrolyte, confirmed through electrochemical evaluations. Additionally, the carbon and C/GT photoanodes were evaluated up to 12 and 125 h, respectively, in contact with an aqueous solution under continuous solar irradiation, demonstrating their electrical and protective capacity in devices with a large active area of >1.1 cm^2^.

## 4 Experimental Section

### Materials

For the synthesis of the perovskite Cs_0.05_(FA_0.9_MA_0.1_)_0.95_Pb(I_0.9_Br_0.1_)_3_, precursors and salts of PbI_2_ (99.99%), PbBr_2_ (>98%), and CsI (>99%) were sourced from TCI Chemicals. The organic cations formamidinium iodide (FAI >99.99%) and methylammonium bromide (MABr >99.99%) were obtained from GreatCell Solar. The solvents DMF and DMSO were acquired from Sigma–Aldrich. For the inorganic HTL materials, copper thiocyanate (CuSCN) and dipropyl sulfide (Sigma Aldrich) were used, along with a 0.2 µm hydrophobic PTFE filters from Sartorius. Polymeric reagents were used, including polymethyl methacrylate (PMMA) in both its components (liquid phase and prepolymerized solid phase) from the Veracril brand by New Stetic, and polyamide (PA) resin. Graphite nanoparticles (Graphite Nanopowder –400 nm, 99.9%) from NanoAmor and carbon black (Acetylene, 50% compressed, 99.9%) from Alfa Aesar were tested for the fabrication of carbon electrodes. A compact, conductive graphite tape (Thermal Interface Sheet, Graphite, 1600 W m^−1^·K, 180 × 115 mm, 0.025 mm, Self‐Adhesive) from Panasonic was utilized. Additionally, a nanoparticulate dispersion of NiFe‐LDH was employed as a catalyst using nickel(II) hexahydrate nitrate (Ni(NO_3_)_2_‐6H_2_O) >99.99% from Alfa Aesar and iron(III) nonahydrate nitrate (Fe(NO_3_)_3_‐9H_2_O) >98% from PanReac AppliChem. Potassium hydroxide (KOH) >85% from EMSURE was used as the electrolyte.

### Synthesis of Triple Cation Perovskite

To obtain the triple cation perovskite precursor, 1.5 m stock solutions of PbI_2_ and PbBr_2_ are prepared in a 4:1 (volume ratio) solution of DMF and DMSO. A quantity of FAI is weighed and added to the corresponding PbI_2_ stock solution with a molar ratio of 1:1.07, respectively, to produce 1.22 m FAPbI_3_ solution. A similar process is followed for 1.22 m MAPbBr_3_ solution, where a quantity of MABr is weighed and added to the PbBr_2_ stock solution with a molar ratio of 1:1, respectively. Finally, both FAPbI_3_ and MAPbBr_3_ solutions are mixed, and 5% of 1.22 CsI solution dissolved in DMSO is added to obtain a perovskite solution with the chemical formula Cs_0.05_(FA_0.9_MA_0.1_)_0.95_Pb(I_0.9_Br_0.1_)_3_. For the deposition of the triple cation perovskite, a static spin‐coating condition of 2 cycles is utilized. The first cycle involves depositing 50 µL of perovskite onto the substrate, followed by spinning at an acceleration and speed of 200 rpm s^−1^ and 1000 rpm, respectively, for 10 s. The second cycle spins at 2000 rpm s^−1^ and 6000 rpm for 20 s, and 150 µL of anisole is dropped as antisolvent with 5 s remaining. Afterward, annealing at 150 °C is performed for 15 min. The process was made according to a previous work.^[^ ^67^ ^]^

### Synthesis of CuSCN

The CuSCN solution, with a concentration of 15 mg mL^−1^, is produced from a diluted copper thiocyanate salt in a filtered solution of dipropyl sulfide. The mixture is stirred magnetically overnight at room temperature to ensure complete dissolution of the salt. The deposition of the HTL layer was performed by spin‐coating 60 µL of solution at 4000 rpm and 4000 rpm s^−1^ for 30 s. After each deposition, an annealing step was performed at 60 °C for 2 min.

### Synthesis of Carbon Electrode

The carbon pastes were carried out with the aim of obtaining a carbon electrode with optimal properties. The process involved mixing polymeric reactants with carbon nanoparticles. The carbon paste was obtained in the following four steps: weighing the carbon materials, weighing the solid prepolymerized PMMA material, adding the liquid monomeric agent (MMA), and subjecting the mixture to magnetic stirring for 15 h for proper homogenization. The carbon electrodes were formed from this paste using blade coating at a sweep speed of 4.16 cm s^−1^ with a gap of 250 and 300 µm for solar cells and photoanodes, respectively.

### Synthesis of NiFe‐LDH

The synthesis of the NiF‐LDH catalyst, which was performed using the procedure found in the previous works,^[^ ^40^, ^41^ ^]^ was corroborated by X‐ray diffraction (XRD) and Raman Spectroscopy. XRD patterns were acquired using a Rigaku MiniFlex 600 equipment. Scans between 2*θ* = 5° and 70° were performed using a step of 0.01°, a speed of 2° min^−1^, a voltage of 40 kV, a current of 15 mA and a Cu‐Kα radiation source (*λ* = 1.5405 Å). Raman scattering was carried out using a Horiba Yvon equipment with 632.81 nm laser excitation. For the exfoliation of nanoparticles and the extraction of the catalyst, a Sonics Vibra‐Cell model VCX130 was utilized for strong sonication. The sonication process lasted for 30 min with an applied amplitude of 70%, resulting in energy outputs of 21.4 kJ.

### Solar Cells Fabrication

The devices were manufactured on glass substrates with ITO (indium‐tin oxide) coatings measuring 2.5 cm x 2.5 cm. Initially, the substrates were washed with neutral soap, rinsed with water, and immersed in three different solvents: DI water, acetone, and isopropanol, where they were subjected to ultrasound for 10 min each. The electron transport layer (ETL) of the device (SnO_2_) was prepared using a two‐step method. First, the cleaned and dried ITO glass substrates were subjected to ultraviolet‐ozone (UVO) treatment for 15 min at 50 °C to enhance surface wettability prior to the first step. This step involved preparing a solution of SnCl₂·5H₂O at a concentration of 15.5 mg mL^−1^, which was deposited using dynamic spin‐coating with settings of 1000 rpm s^−1^ for acceleration, 3000 rpm for speed, and 30 s for time, applying a volume of 80 µL. Subsequently, the samples were annealed at 100 °C for 10 min and then at 180 °C for 1 h. The second step consisted of a chemical bath prepared with 250 mL of DI water, 3.75 g of urea, 62.5 µL of mercaptopropionic acid, 3.75 mL of HCl, and 675 mg of SnCl₂·2H₂O. The substrates were immersed in this chemical bath and heated in an oven at 70 °C for 3 h before being rinsed with DI water to proceed with drying and labeling. Afterward, the triple cation perovskite, CuSCN and carbon electrode were deposited as mentioned above. To achieve the structure shown in Figure 1a, with a carbon electrode and an area of 0.085 cm^2^ for each of the 5 electrodes (h^+^ extraction) and, for the counter‐electrode (e^−^ extraction), a mask is used that adheres to the CuSCN layer and defines the active areas that are subsequently covered by carbon while maintaining the processing parameters. Once the carbon electrode is deposited, the mask is removed and allowed to dry at room temperature.

### Photoanodes Fabrication

For the fabrication of the photoelectrodes and obtaining the structure shown in Figure 3a, the same process as that of solar cells is followed. However, for the deposition of the carbon electrode, the mask that adheres to the CuSCN layer is changed. The modification consists of creating only a large area electrode (1.2–1.3 cm^2^) located at the center along with the counter electrode. When the mask is removed, a new mask is applied, revealing only the active areas coated with carbon. For devices using graphite tape, the adhesive is removed by immersing it in acetone and then detaching it. When the graphite sheet is removed from the adhesive, it is cut in such a way that it achieves an area slightly larger than 1.3 cm^2^ and is placed over the large carbon electrode (see Figure 3b), which is then sealed with the new mask, exposing only the active areas of graphite and carbon in this case. Subsequently, for both cases, the deposition of the catalyst on the large area electrode of both carbon and graphite is carried out while preventing the catalyst from depositing on the carbon counter electrode. This was done as follows: the coupling of the NiFe‐LDH catalyst onto the carbon electrode and graphite tape was performed by spray coating an aqueous solution with concentration of 18 mg mL^−1^ using a Meinhard concentric quartz spray nozzle with an air pressure of 4 MPa for a flow rate of 0.5 mL min^−1^, while the substrate was kept at a temperature of 100 °C during the catalyst deposition, with a distance from the nozzle to the base of the heating plate of 13 cm. The catalyst was deposited at 12 deposition cycles. Deposition cycles were performed under static conditions, with 10 s of deposition followed by 15 s of annealing to allow the solvent evaporation. After this, all samples were placed in a glovebox for subsequent evaluation.

### Morphological Characterization

It was carried out using a dual beam field emission scanning electron microscope (FIB – FESEM) from Thermo Fisher Scientific. Backscattered electron (T1) and secondary electron (T2) detectors were employed to obtain images from both top views and cross‐sections. A Keithely 4200A‐SCS system coupled with a solar simulator was used to assess the photovoltaic parameters of the devices. An Ossila class AAA solar simulator was also used, designed for small‐area cells, featuring LED lights with a spectral coverage greater than 80% for the direct evaluation of green hydrogen generation devices. X‐ray diffraction (XRD) analysis was employed to evaluate the morphological and structural characteristics of the perovskite films and the hole transport layer (HTL). Photoluminescence (PL) properties of the films were assessed using a VARIAN CARY Eclipse device.

### Electrochemical Measurements

These were conducted with a three‐electrode system utilizing a custom‐made 3D‐printed ABS chemical reactor. A 6 mm diameter graphite rod from PalmSens served as the counter electrode, alongside a home‐made Hg/HgO reference electrode, both immersed in a 1 m KOH electrolytic medium. Linear sweep voltammetry (LSV), chronoamperometry, and electrochemical impedance spectroscopy (EIS) were performed using a Gamry 600 potentiostat. The LSVs were acquired with a step of 2 mV and a scan rate of 20 mV s^−1^, ranging from 0.25 to 1.1 V. Chronoamperometry measurements were taken at current densities of 10 mA cm^−2^ for 30 min and 12 h, while EIS measurements were conducted close to the open circuit potential (OCP), oscillating between 1000 kHz and 0.1 Hz with 10 points per decade.

### In Situ Raman Spectroscopy

Raman scattering experiments were performed using a Horiba Yvon system equipped with a 632.81 nm laser excitation source. Measurements were carried out both before and during the OER, using the same counter and reference electrodes employed in the electrochemical experiments. To enable direct laser access to the C/NiFe‐LDH working electrode, a custom mini‐reactor without a cover was used, allowing for in situ measurements under controlled polarization conditions.

### Three‐Electrode Set‐Up

The efficiency of these systems is determined by 2 current densities versus potential (*J–V*) curves of the photoelectrodes, and the operating point is the short‐circuit current density of the photoanodic‐photocathodic cell, corresponding to the current density at 0 V of polarization.^[^ ^24^, ^25^ ^]^ The efficiency is achieved when such systems do not require an additional voltage to facilitate the water splitting reaction, resulting in a nonpolarized system resulting in which uses Equation (2).

(2)

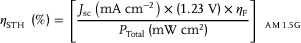




Conversely, when the systems are measured under a certain applied bias, a similar expression to Equation (2) is used, known as the applied bias photon‐to‐current efficiency (ABPE), this represents the ratio of incident solar energy converted into hydrogen energy under specific polarization conditions, as expressed in Equation (3).^[^ ^24^, ^25^ ^]^

(3)

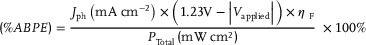




## Conflict of Interest

The authors declare no conflict of interest.

## Supporting information



Supporting Information

## Data Availability

The data that support the findings of this study are available from the corresponding author upon reasonable request.
