# Supplementary material for: Full‐Solution Processed Halide Perovskite Photoanodes with Carbon/NiFe‐LDH Protection for Efficient Photoelectrochemical Water Oxidation
Source: Small. 2025 Jun 12;21(31):2412713. doi: 10.1002/smll.202412713 (PMC12332817; doi:10.1002/smll.202412713)
Supplement: Supplementary file 1 — Supporting Information [file SMLL-21-2412713-s001.docx]

**Full-solution processed halide perovskite photoanodes with Carbon/NiFe-LDH protection for efficient photoelectrochemical water oxidation**

Carlos A. Velásquez ^a^, Juan J. Patiño ^a^, Kevin Ballestas ^a^, Franklin Jaramillo^a^, Juan F. Montoya ^a,b^, Daniel Ramírez ^a *^

^a^ Centro de Investigación, Innovación y Desarrollo de Materiales – CIDEMAT, Universidad de Antioquia UdeA, Calle 67 No. 52-21, Medellín, Colombia.

^b^ Grupo de Catalizadores y Adsorbentes (CATALAD), Instituto de Química, Facultad de Ciencias Exactas y Naturales, Universidad de Antioquia UdeA, Medellín 50001, Colombia

*Corresponding author: estiben.ramirez@udea.edu.co

**Table S1.** Comparison of perovskite-based photoanodes in the water splitting reaction reported in the literature.

| **Photoanode structure (OEC/HTL/perovskite/ETL/ substrate)** | **Area [cm^2^]** | **Electrolyte [pH]** | **Photocurrent density [mA/cm^2^]** | **Onset Potential [V]** | **ABPE^a)^ [%]** | **Stability [h]** | **Ref** |
| --- | --- | --- | --- | --- | --- | --- | --- |
| NiFe-LDH/Carbon/CuSCN/ Cs_0.05_(FA_0.9_MA_0.1_)_0.95_Pb(I_0.9_Br_0.1_)_3_/SnOx/ITO | 1.1 | 1M KOH (pH:14) | 11.71 @ 1.23 V_RHE_^b)^ | 0.4 V_RHE_ | 4.57% @ 0.64V_RHE_ | 12 | This work |
| NiFe-LDH/Graphite tape/Carbon/CuSCN/ Cs_0.05_(FA_0.9_MA_0.1_)_0.95_Pb(I_0.9_Br_0.1_)_3_/SnOx/ITO | 1.1 | 1M KOH (pH:14) | 18.07 @ 1.23 V_RHE_ | 0.37 V_RHE_ | 8.5% @ 0.65V_RHE_ | >120 |  |
| Perovskite Photoanodes | | | | | | | |
| NiFeOOH/Ni foil/Ag/Au /SpiroOMeTAD/FAPbI_3_/TiO_2_/FTO/Cu wire | 0.25 | 1M KOH (pH:14) | 22.82 @ 1.23 V_RHE_ | 0.41 V_RHE_ | 7.93% @ 0.765 V_RHE_ | 75 | ^[1]^ |
|  |  |  |  |  |  |  |  |
|  |  |  |  |  |  |  |  |
| IrOx/Conductive adhesive-barrier (CAB)/Au/SpiroOMe TAD/ FA_0.97_MA_0.03_PbI_3_/SnO_2_/FTO | - | 0.5M H_2_SO_4_ (pH 0) | 19.8 @ 1.23 V_RHE_ | 0.37 V_RHE_ | 11.3% @ 0.66 V_RHE_ | 6 | ^[2]^ |
|  |  |  |  |  |  |  |  |
|  |  |  |  |  |  |  |  |
| Ni/Graphite sheet /SpiroOMeTAD/FACsPbIBr_3_/TiO_2_ /FTO/Cu wire | 0.14 | 1M KOH (pH:14) | 17.4 @ 1.23 V_RHE_ | 0.60 V_RHE_ | 5.82% @ 0.8 V_RHE_ | 40 | ^[3]^ |
|  |  |  |  |  |  |  |  |
|  |  |  |  |  |  |  |  |
| Carbon/Ag paint/Carbon/ MAPbI_3_/TiO_2_/FTO | - | 1M KOH (pH:14) | 12.4 @ 1.23 V_RHE_ | 0.80 V_RHE_ | 0.88% @ 1.1 V_RHE_ | 12 | ^[4]^ |
|  |  |  |  |  |  |  |  |
|  |  |  |  |  |  |  |  |
| FeNi(OH)x/Ni foil- Ag/Au/SpiroOMe TAD/FAMACsPbIBr/TiO_2_/FTO | 0.5 | 0.5M NaOH (pH 13.7) | 11.6 @ 1.23 V_RHE_ | 0.39 V_RHE_ | - | 13.1 | ^[5]^ |
|  |  |  |  |  |  |  |  |
|  |  |  |  |  |  |  |  |
| Ni/Au/SpiroOMeTAD/ MAPbI_3_/TiO_2_/FTO | 0.122 | 0.1M Na_2_S (pH 12.8) | 10 @ 0.95 V_RHE_ | 0.35 V_RHE_ | - | 0.2 | ^[6]^ |
|  |  |  |  |  |  |  |  |
|  |  |  |  |  |  |  |  |
| IrOx/Graphite sheet/m- Carbon/Ag/CsPbBr_3_/TiO_2_/FTO | - | H_2_SO_4_ + KNO_3_ (pH 2.5) | 3.8 @ 1.23 V_RHE_ | 0.60 V_RHE_ | - | 7.8 | ^[7]^ |
|  |  |  |  |  |  |  |  |
|  |  | 0.1M KNO_3_ (pH 7) | 2.5 @ 1.23 V_RHE_ | - |  | 23 |  |
|  |  |  |  |  |  |  |  |
|  |  | KOH + KNO_3_ (pH 12.5) | 2.0 @ 1.23 V_RHE_ | - |  | 34 |  |
|  |  |  |  |  |  |  |  |
| MOF derived Co_3_O_4_ - N-doped carbon/CsPbBr3/FTO | - | 1M NaOH (pH 14) | 2.5 @ 1.23 V_RHE_ | 0.09 V_RHE_ | 1.52% @ 0.45 V_RHE_ | 24 | ^[8]^ |
|  |  |  |  |  |  |  |  |
|  |  |  |  |  |  |  |  |
| Ni/Ag/SpiroOMeTAD/TEA modified MAPbI_3_/TiO_2_/FTO | 0.063 | 0.1M Na_2_S (pH 13) | 2.08 @ 0.96 VAg/AgCl | (0.60 V_Ag/AgCl_) | - | 0.50 | ^[9]^ |
|  |  |  |  |  |  |  |  |
|  |  |  |  |  |  |  |  |
| MAPbI_3_/TiO_2_NR/TiO_2_/FTO | 1 | 57 wt% Aq. HI + MAPbI_3_ | 1.75 @ 0.4 V_RHE_ | - | - | 8 | ^[10]^ |
| Ni/Ag/SpiroOMeTAD/MAPbI_3_ (TBA modified)/FTO | 0.063 | 0.1M Na_2_S (pH 13) | 0.42 @ 0.96 V_RHE_ | - | - | - | ^[9]^ |
| NiFeOOH+Graphite porous /Adhesive/ Graphite less porous/Adhesive/Printed-Carbon/SpiroOMeTAD/CsPbBr_3_/TiO_2_/FTO | 0.03 – 0.28 – 1.1 | 1M NaOH (pH 14) | 8.1 @ 1.23 V_RHE_ | 0.4 V_RHE_ | - | >100 | ^[11]^ |
| NiFeOOH/Ni/Au/FAMAPbI3 | - | 1M KOH (pH 14) | 24 @ 1.23 V_RHE_ | 0.56 V_RHE_ | 9.16% @ - | 48 | ^[12]^ |
| NiFeOOH**/Ni/Protective sheet/Adhesive/Printed carbon/** CsPbBr_3_/SnO_2_/FTO/Glass | 0.197 | 1M NaOH (pH 14) | 8.0 @ 1.23 V_RHE_ | 0.4 V_RHE_ | 2.45% @ 0.7 V_RHE_ | 210 | ^[13]^ |
| FTO/TiO_2_/CsPbBr_3_/WCTG | 0.2 | 0.1 M KNO_3_ (pH 14) | 4.6 @ 1.23 V_RHE_ | 0.5 V_RHE_ | - | 5 | ^[14]^ |
| Ni/C/Ag/C/MAPbI_3_ /m-TiO_2_ | 0.08 | 1M NaOH (pH 14) | 16.55 @ 1.23 V_RHE_ | 0.44 V_RHE_ | 4.57% @ 0.79 V_RHE_ | 30.9 | ^[15]^ |
| Photoanodes (Other materials) | | | | | | | |
|  |  |  |  |  |  |  |  |
| TiO_2_/ZnO/CZTS | 0.2 | 0.1M Na_2_SO_4_ (pH 6.8) | 15.05 @ 1.23 V_RHE_ | 0.138 V_RHE_ | 6.9% @ 0.64 V_RHE_ | 1 | ^[16]^ |
|  |  |  |  |  |  |  |  |
|  |  |  |  |  |  |  |  |
| NiFeLDH/Ni foil/Organic | 0.5 | 1M NaOH (pH 13.6) | 15.1 @ 1.23 V_RHE_ | 0.55 V_RHE_ | 4.33% @ 0.82 V_RHE_ | 10 | ^[17]^ |
|  |  |  |  |  |  |  |  |
|  |  |  |  |  |  |  |  |
| NiFeLDH/n+p Si | 1 | 1M KOH (pH 14) | 37 @ 1.23 V_RHE_ | 0.78 V_RHE_ | 4.3% @ 1.02 V_RHE_ | 68 | ^[18]^ |
|  |  |  |  |  |  |  |  |
|  |  |  |  |  |  |  |  |
| NiFeO_x_/B:C_3_N_4_/Mo:BiVO_4_ | 1 | 0.5M KPi + 0.1M Na_2_SO_3_ (pH 7) | 5.93 @ 1.23 V_RHE_ | 0.15 V_RHE_ | 2.67% @ 0.54 V_RHE_ | 10 | ^[19]^ |
|  |  |  |  |  |  |  |  |
|  |  |  |  |  |  |  |  |
| TiO_2_/Black BiVO_4_ | - | 0.5M KPi (pH 7) | 6.12 @ 1.23 V_RHE_ | 0.21 V_RHE_ | 2.50% @0.56 V_RHE_ | 100 | ^[20]^ |
|  |  |  |  |  |  |  |  |
|  |  |  |  |  |  |  |  |
| IrCoO_x_/Ta_3_N_5_ | - | 1M NaOH (pH 13.6) | 12.1 @ 1.23 V_RHE_ | 0.65 V_RHE_ | 2.50% @ 0.9 V_RHE_ | 1.25 | ^[21]^ |
|  |  |  |  |  |  |  |  |
|  |  |  |  |  |  |  |  |
| NiCoFe-Bi/Ta_3_N_5_ | 0.94 | 1M NaOH (pH 13.6) | 9.3 @ 1.23 V_RHE_ | 0.47 V_RHE_ | 3.46% @0.77 V_RHE_ | 2.67 | ^[22]^ |
|  |  |  |  |  |  |  |  |
|  |  |  |  |  |  |  |  |

^a)^ ABPE: applied polarization efficiency from photon to current; ^b)^V_RHE_: V vs Reference Hydrogen Electrode.

**Triple cation Perovskite and hole transport layer (HTL) films characterization**


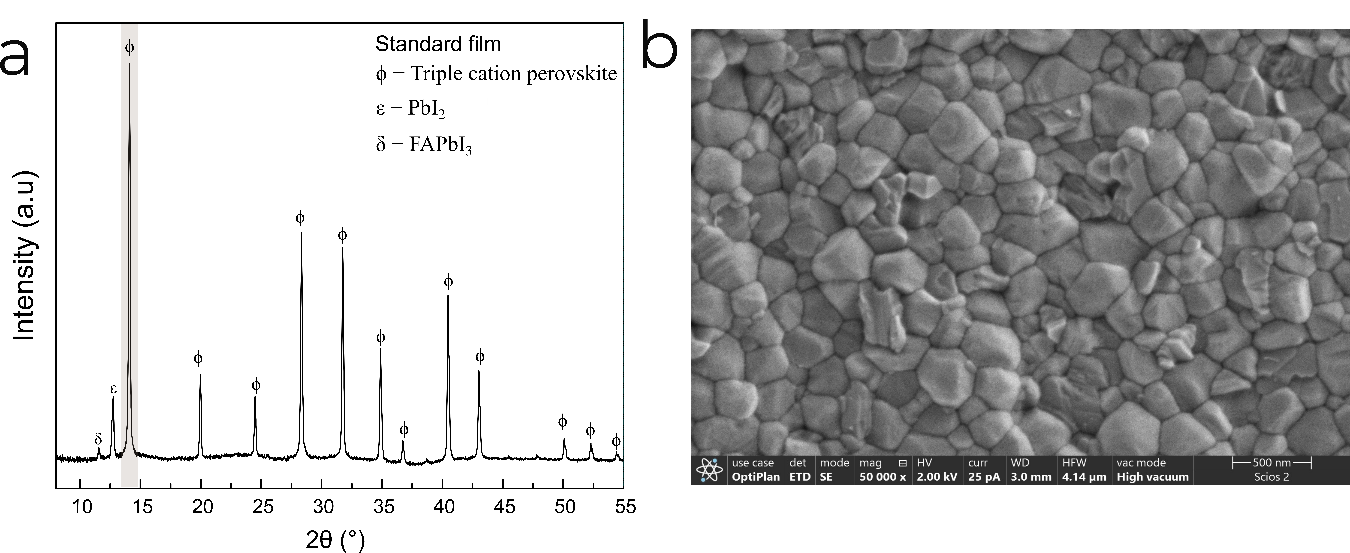


**Figure S1.** Triple cation perovskite layer **a)** XRD and **b)** SEM – Top view at 50000X.

**Figure S1a** shows the x-ray diffraction (XRD) pattern of the triple cation perovskite having a main peak at a 2θ = 14 °, associated with the active α-phase of the triple cation perovskite, specifically at 2θ = 14.34, 20.4, 24.84, 28.7, 32.2, 35.3, 40.1, and 43.6 °.^[23,24]^ Two additional peaks at 11.6 and 12.7 ° correspond to the photoinactive hexagonal δ-phase of FAPbI_3_ and the cubic phase of PbI_2_, respectively. Both peaks are typical for the mixed composition of perovskite, indicating an incomplete conversion of FAPbI_3_ perovskite within the photoactive black phase. **Figure S1b** shows a top-view scanning electron microscopy (SEM) image of the triple cation perovskite film.

| **Perovskite** | **1 CuSCN** | **2 CuSCN** | **3 CuSCN** |
| --- | --- | --- | --- |
| 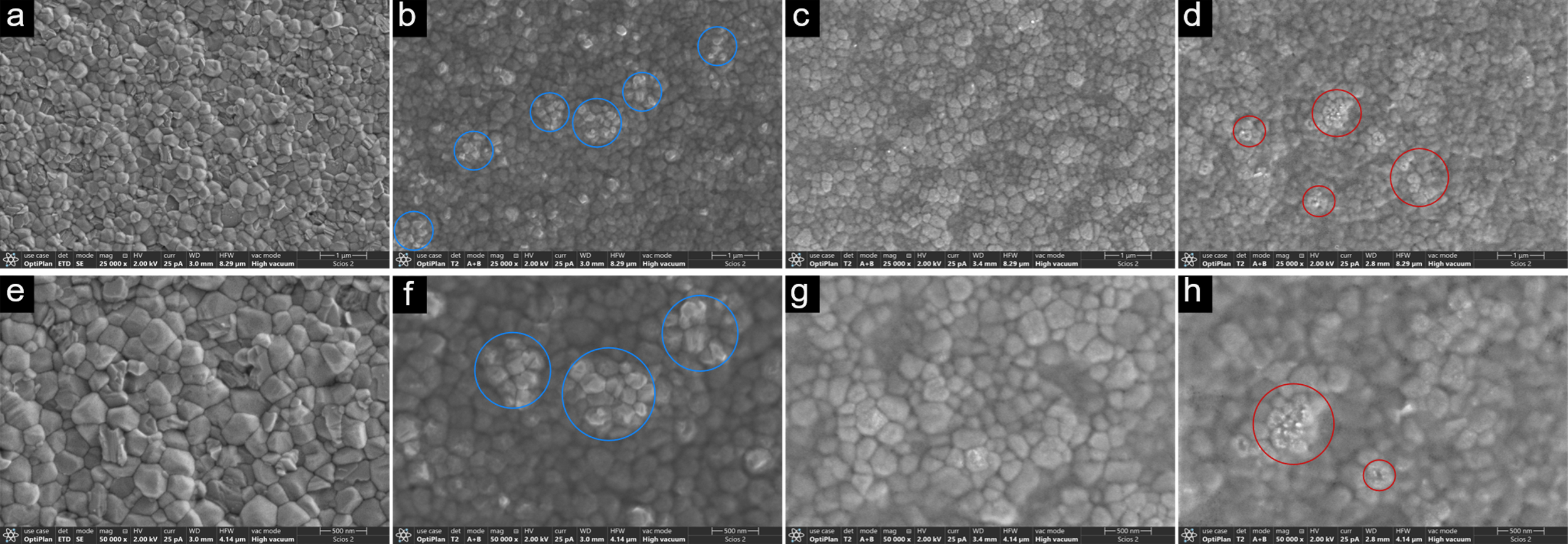 | | | |

**Figure S2.** Top view SEM images using secondary electrons. Triple cation perovskite grains at magnifications of **a)** 25,000X and **e)** 50,000X. CuSCN layer fabricated using **b, f)** 1, **c, g)** 2 and **d, h)** 3 deposition cycles at 25000 and 50000X magnifications, respectively.

**Figures S2a and S2e** show the initial morphology of the triple cation perovskite layer without CuSCN coating, where it is possible to identify the granular structure, with a typical size within the range of 300 to 400 nm.^[24]^ In **Figures S2b and S2f** it can be observed from the images obtained of CuSCN coverage on the grains that a partial coverage was achieved for one deposit of the CuSCN solution on top of the perovskite film, where the spots with worst coverage are shown in the blue circles. However, with two deposits of CuSCN, greater and more homogeneous coverage is evident, allowing the formation of a porous structure that covers a larger number of perovskite grains, as seen in **Figures S2c and S2g**. Finally, the structure with three deposition cycles in **Figures S2d and S2h** shows a complete coverage of the grains. However, it is noted that some spots have aggregates of CuSCN, as shown in the red circles, indicating that there is not a completely homogeneous film formation. This likely impacts the optical and electronic behavior of the device, as evidenced in the photoluminescence study below.


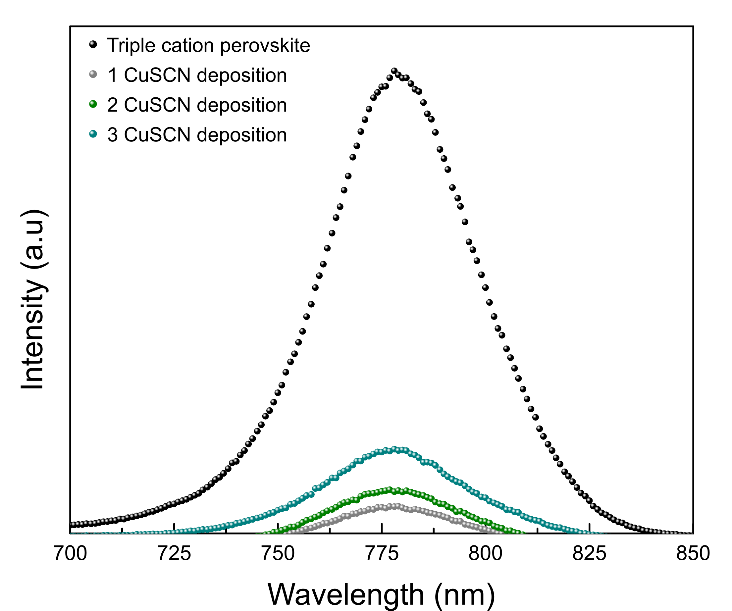


**Figure S3.** Photoluminescence of triple cation perovskite films without any CuSCN and with CuSCN layers obtained using 1, 2 and 3 deposition cycles.

As a complement to morphological measurements, the photoluminescence of the resulting perovskite film processed under the same 4000 rpm conditions displays high energy absorption and emission associated with the pronounced peak observed at a wavelength of 775 nm. On the other hand, photoluminescence evaluation was conducted by varying the number of deposition cycles of CuSCN over a triple cation perovskite film, aiming for a homogeneous layer with an appropriate thickness for the hole transport layer (HTL). Visually, the film did not show a significant change. Noting that the CuSCN solution was developed with the highest possible concentration in mg/mL for achieving a dilution, layers of CuSCN were deposited on top of one another since there would be no removal of the underlying layer. As seen in **Figure S3**, the best quenching condition was observed with one deposit. However, this showed minimal difference compared to the film with two deposits. Conversely, the film with three deposits experienced decreased quenching, indicating inadequate charge capture generated in the semiconductor under this condition.

**Photovoltaic performance of carbon based – perovskite solar cells (C-PSCs)**

Devices with 1, 2, and 3 cycles of CuSCN deposition were fabricated and their performance is shown in **Figure S4**.


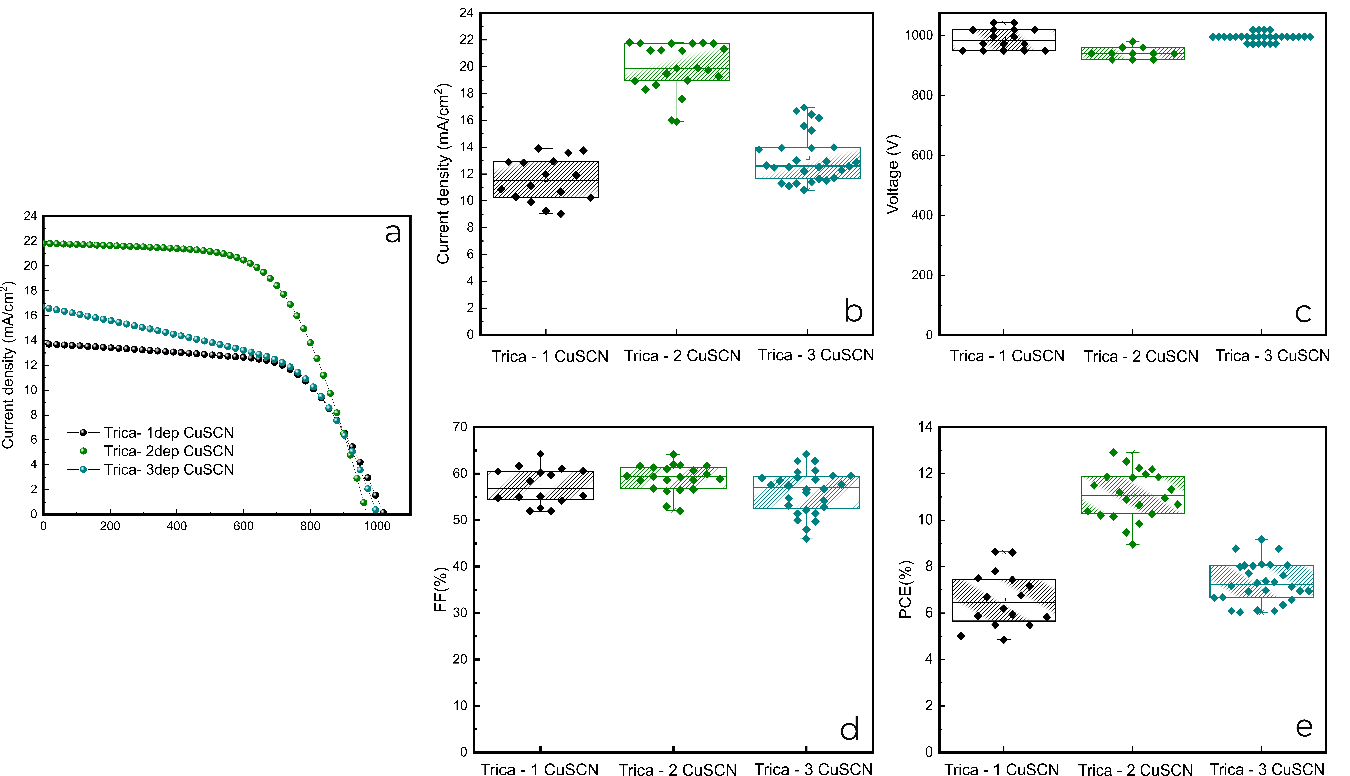


**Figure S4.** Photovoltaic parameters of the solar cell with a carbon electrode after 1, 2, and 3 deposition cycles of CuSCN with n:36. **a)** IV curve, **b)** current density (mA/cm²), **c)** voltage (V), **d)** FF (%), and **e)** PCE (%). Statistical data (b-e) correspond to 12 replicas (n=12) of each device configuration.

**Figure S4a** shows the JV curves for each evaluated condition, in which a peak in short-circuit current (J_sc_) is observed for the HTL layer made from two deposition cycles of CuSCN. This trend is confirmed in **Figure S4b**, where the highest average current is achieved with the 2-cycles condition. However, for the 3-cycles condition the J_sc_ is lower, while the lowest current was recorded for the 1-cycle condition. **Figure S4c** shows that the voltage attained for each condition does not show any significant change. The same trend is observed in **Figure S4d** for the FF, where the average for each condition remains relatively unchanged, indicating that the differences in PCE (**Figure S4e**) between the different conditions are mainly due to the J_SC_. Based on this, the optimal condition is the one with 2 deposition cycles. In addition, it is noteworthy that for this type of device, a solar cell was obtained with a maximum efficiency of 13%, while the average PCE was 10.86 % as shown in **Figure S4e**.

In Situ Raman Spectroscopy:


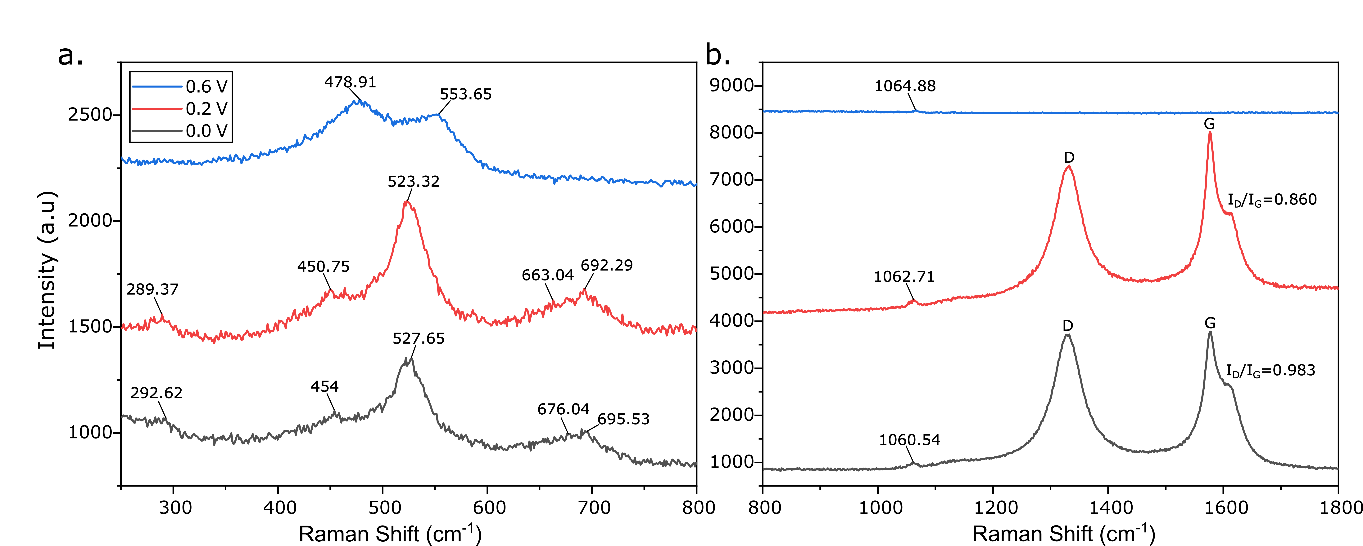


**Figure S5.** In situ Raman spectra of the Carbon/NiFe-LDH electrode under applied potentials relevant to the OER (0.0, 0.2, and 0.6 V), shown over two spectral regions: **(a)** 250–800 cm^-1^ and **(b)** 800–1800 cm^-1^.

**Figure S5**. presents in situ Raman spectroscopy measurements used to investigate the chemical structure transformations of the catalyst under polarization conditions, performed before (0.0 and 0.2 V) and after (0.6 V) the onset of the oxygen evolution reaction (OER). Figure S10a shows the Raman spectrum of the NiFe-LDH deposited on a carbon electrode at 0.0 V, revealing bands at 454, 527.65, 676.04, and 695.53 cm⁻¹. These are assigned to α-Ni(OH)₂, γ-FeOOH, and γ-Fe₂O₃, respectively.^[25,26]^

Upon applying a mild voltage (0.2 V), prior to the OER onset, the Raman signals become more pronounced, and a redshift is observed for the same vibrational modes (450.75, 523.32, 663.04, and 692.29 cm⁻¹). This redshift is attributed to changes in the vibrational modes due to the applied bias, hydration of the α-Ni(OH)₂, γ-FeOOH, and γ-Fe₂O₃ phases, and partial strain relaxation.^[27,28]^ In addition, the bands at 292.62 and 289.37 cm⁻¹ observed at both 0.0 and 0.2 V correspond to E-type vibrations, associated with M–O in-plane motions within the α-Ni(OH)₂ layers, where atomic displacements involve degenerate vibrational components.^[29,30]^ At 0.6 V, the onset potential for the OER has been exceeded, leading to the formation of γ-NiOOH, as indicated by Raman bands blue-shifted to 478.91 and 553.65 cm⁻¹. These shifts reflect the oxidation of Ni²⁺ (present in α-Ni(OH)₂) to Ni³⁺/Ni⁴⁺ species, which are highly active in the OER process. The combination with the iron species further enhances catalytic activity. However, the vibrational signals of the iron species are weaker and may overlap with the more intense NiOOH signals.^[25,26]^

Figure S10b shows the Raman spectra in the range of 800–1800 cm⁻¹. At 0.0 V, a distinct band is observed at 1060.54 cm⁻¹, corresponding to CO₃²⁻ species located in the interlayer region of the catalyst.^[26]^ This vibration also appears at 1062.71 cm⁻¹ and 1064.88 cm⁻¹ for the spectra at 0.2 V and 0.6 V, respectively. Notably, the intensity of this band at 0.6 V is lower than in the other cases, likely due to structural expansion of the interlayer spacing under OER conditions, which facilitates the release of CO₃²⁻ species.

The D and G bands of the carbon support are clearly visible at 0.0 and 0.2 V. The G band exhibits a double peak structure, attributed to the coexistence of crystalline graphite and amorphous carbon domains.^[31]^ Additionally, the I_D_/I_G_ ratio decreases from 0.983 to 0.860 upon mild polarization (0.2 V), suggesting partial ordering of the carbon structure, likely due to surface reorganization or defect passivation. This structural evolution may enhance the electrical conductivity of the carbon support by improving sp² domain connectivity and reducing charge carrier scattering,^[32]^ thereby facilitating more efficient charge transfer at the carbon/NiFe-LDH interface. Furthermore, this improvement in the carbon lattice, when combined with the high conductivity of the GT could reduce the overall resistivity of the system.

In the 0.6 V spectrum, the intensity of the D and G bands decreases significantly. This reduction is likely due to the formation of a NiOOH/FeOOH overlayer. During anodic polarization, the NiFe-LDH catalyst transforms into NiOOH and FeOOH phases, forming a dense oxyhydroxide layer on the surface.^[25]^ This layer can physically block or optically interfere with the Raman excitation and scattered light, effectively shielding the underlying carbon substrate. As a result, the characteristic D and G bands are attenuated or disappear, not due to the degradation of the carbon itself, but because it becomes spectroscopically inaccessible beneath the active catalyst layer.

**Superficial interaction of protective materials**


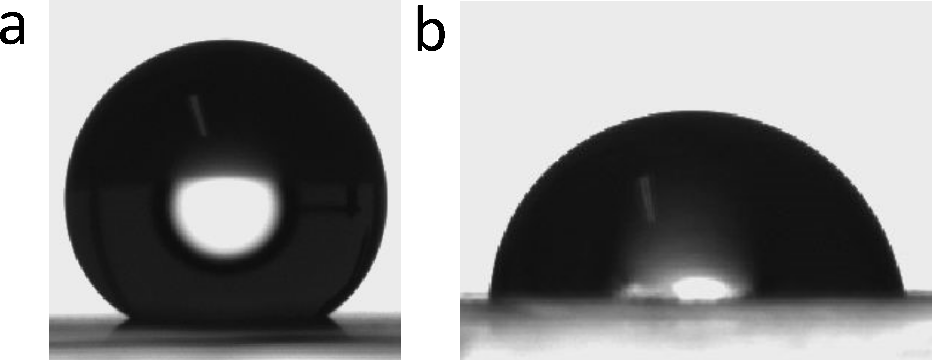


**Figure S6.** Contact angle of **a)** Carbon electrode **b)** Graphite tape.

In **Figure S6a.** it is clearly observed that the carbon electrode exhibits greater hydrophobicity compared to the graphite tape (**Figure S6b**). This result provides an indication of the advantages that the carbon electrode may offer over the commercial electrode (graphite tape) when subjected to high humidity environmental conditions. The repulsion interaction of the standard electrode with ambient moisture will be stronger, thereby inhibiting the perovskite-water interaction to a greater extent. On the other hand, it is important to note that the graphite tape is completely compact, and despite having lower hydrophobicity, any liquid interacting with the devices will not penetrate to the internal layers, making it the better choice in terms of protection. However, the significant issue with this material is its composition: the graphite is adhered to a non-conductive tape, necessitating an additional processing step to utilize them in the devices.

**Assembly and evaluation of photoanode devices.**

| 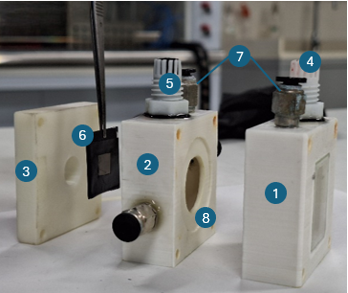 |
| --- |

**Figure S7.** Disassembled chemical reactor with each of its parts: **1.** counter electrode compartment, **2.** working electrode compartment, **3.** support for the device to be evaluated, **4.** counter electrode inlet, **5.** reference electrode inlet, **6.** representation of the photoanode, **7.** gas outlets, and **8.** space for gas separation membrane.

The photoelectrode evaluation was carried out using a measurement system for green hydrogen generation via photoelectrochemical devices as shown in the **Figure S7.** This prototype of PEC systems is the result of an iteration process where several versions were tested leading to design changes to optimize signal acquisition for proper characterization and enhance gas generation. The setup is shown in **Figure S8**.


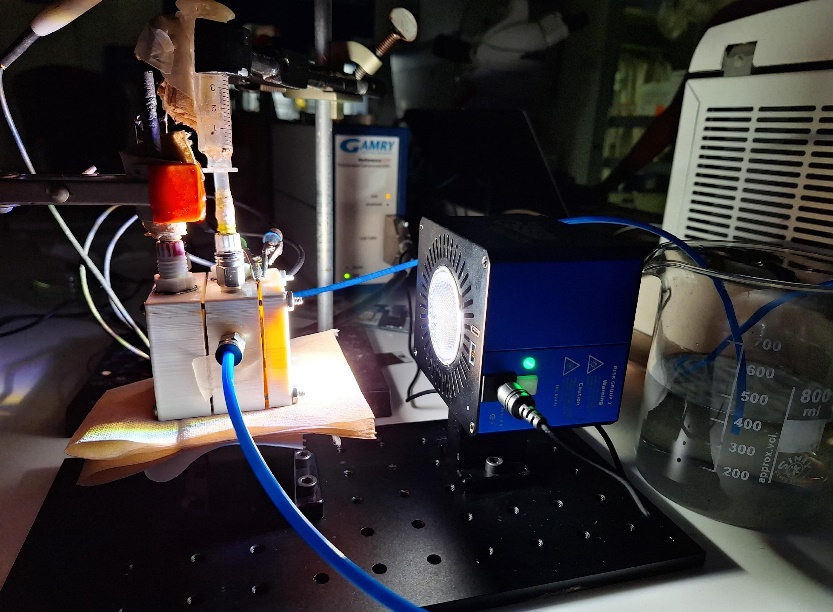

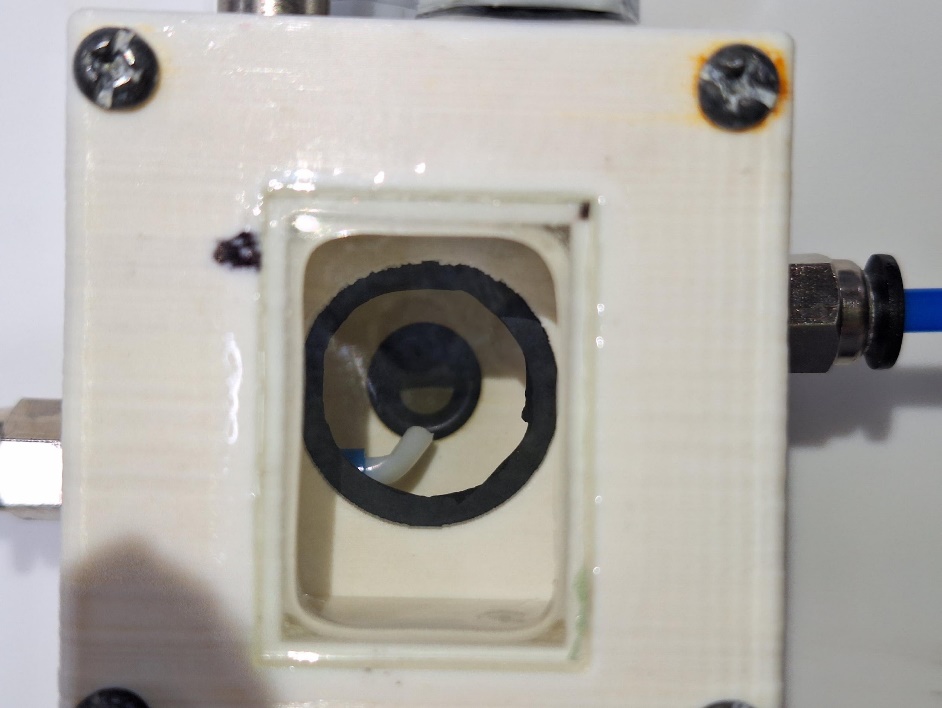


**Figure S8.** Measurement system for green hydrogen generation via photoelectrochemical devices.

For the fabrication of the devices, silver tape was employed to extract electrons that pass to the external system via the potentiostat connection, while adhesive vinyl was used to define the exposed area in the chemical reactor. The configurations used to fabricate the photoelectrochemical devices are shown in **Figure S8**.

| *CE Photoelectrode* | | *GT Photoelectrode* | |
| --- | --- | --- | --- |
| a. | 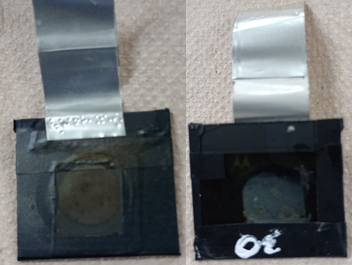 | b. | 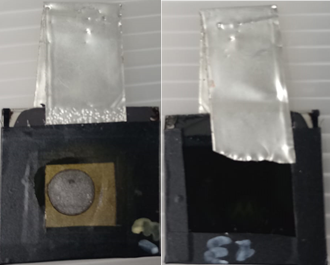 |

**Figure S9.** Front (left) and back (right) side after evaluation of photoelectrochemical devices. Configurations with **a)** carbon/NiFe-LDH and **b)** carbon/GT/NiFe-LDH.

In the devices, the reaction of the catalytic system with the electrolyte is evidently delimitated by the circular active area. For the system with carbon/NiFe-LDH, **Figure S9a** shows partial degradation of the perovskite on the backside, indicated by the white coloration, signaling structural disintegration after 12 h of evaluation. However, for the carbon/GT/NiFe-LDH system (see **Figure S9b**), no degradation is observed after evaluation. This is because the graphite tape added to the structure is completely compact, preventing the electrolyte from penetrating the interior of the structure.


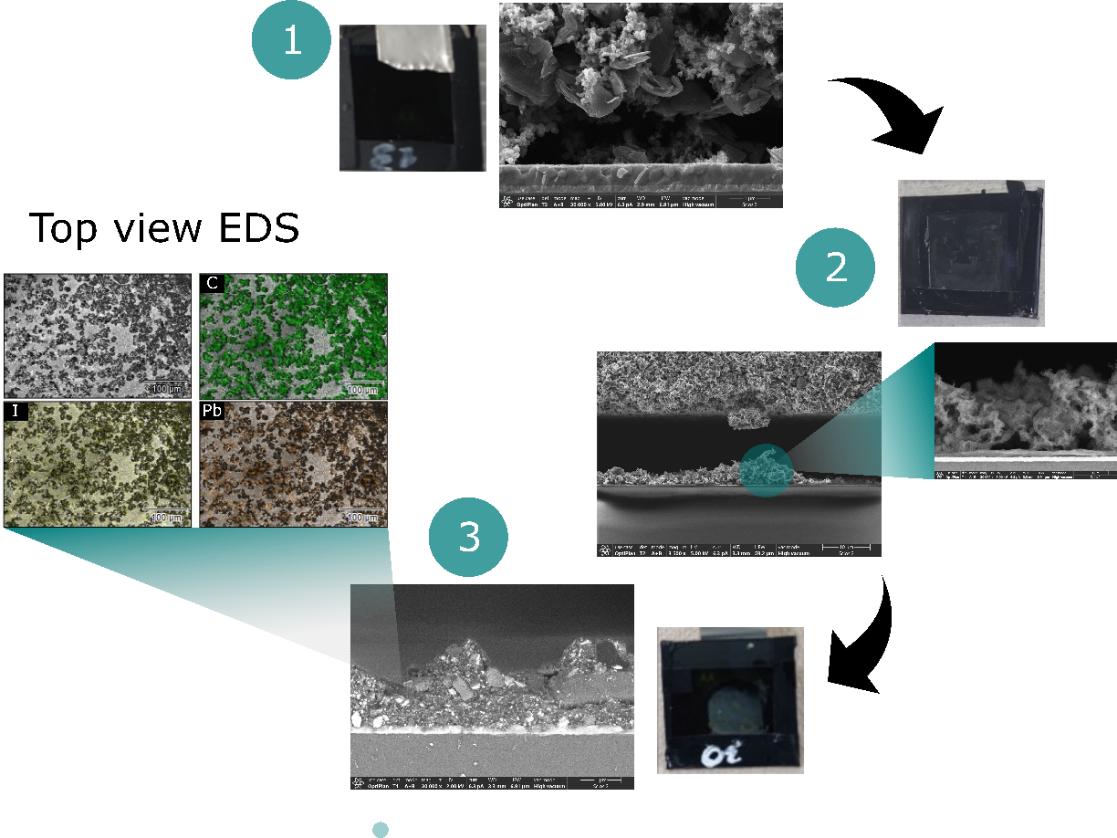


Figure S10. Photoelectrochemical device degradation process.

**Figure S10** systematically illustrates the degradation process of the Cs₀.₀₅(FA₀.₉MA₀.₁)₀.₉₅Pb(I₀.₉Br₀.₁)₃ perovskite under operational conditions. This analysis combines macroscopic photographs, cross-sectional SEM imaging, and EDS elemental mapping. The degradation mechanisms are as follows: the stage 1 shows a pristine device used for solar-driven hydrogen production with intact device under optimal conditions. The cross-sectional SEM image reveals a uniform, well-integrated layer structure with no signs of degradation, representing stable operation. The stage 2. Both device photograph and the cross-sectional SEM image reveal the onset of degradation through the physical detachment of the carbon layer. The perovskite film no longer exhibits its characteristic granular morphology; instead, a completely amorphous layer is observed. This delamination compromises the integrity of the carbon electrode, leaving some areas partially exposed and others covered with remnants of highly porous carbon. These residual fragments, resulting from incomplete delamination, allow direct exposure of the underlying functional layers to the electrolyte. In addition to the structural damage, this process leads to a significant loss of electrical contact, which further accelerates device degradation. The stage 3 evidence an ion migration and structural collapse. In this phase, SEM imaging shows structural disruption and deterioration of the active layers. The photograph reveals discoloration and transparency, suggesting perovskite decomposition. These changes are associated with the migration of iodide and lead ions into adjacent layers, contributing to material destabilization and performance loss. Finally, The EDS image a complete decomposition confirmed by EDS mapping that presents EDS elemental maps highlighting the spatial distribution of key elements. Bright zones for Pb and I appear throughout the top view area, indicating significant ion migration. Carbon is confined to black and irregular areas, and the presence of ITO and glass was evidenced by means top view EDS as large areas of high brightness (Not shown). This confirms that the active perovskite and electrode layers have fully degraded, allowing the direct interaction of electrons by means SEM with the ITO and glass substrate the latter can be seen due to the decomposition process, severely compromising device function.

**Electrochemical characterization**

The parameters obtained from the equivalent circuit modeling are shown in **Table S2** and the respective equivalent circuit of each system are shown in Figure 5.

**Table S2**. Electrical parameters fitted of the experimental Nyquist plot with the equivalent circuit in the **Figure 5**. The resistance parameters were obtained by the **Equation S1** and the electrochemical impedance for the RC circuit was calculated in terms of the CPE as described by **Equation** **S2**.^[2]^

| Parameter | C/NiFe - LDH | C/GT/NiFe - LDH |
| --- | --- | --- |
| Rs (Ohm*cm^2^) | 62.43 | 41.81 |
| Cct1 (F/cm2) | 6.32E-05 | 2.94E-04 |
| Rct1 (Ohm*cm^2^) | 401.00 | 45.24 |
| Cct2  (F/cm^2^) | 5.99E-04 | 1.49E-02 |
| Rct2 (Ohm*cm^2^) | 579.80 | 201.8 |
| 𝑄_𝐷𝑖𝑓𝑓 (S*s^n^) | 3.71E-02 | 4.13E-02 |
| B_𝐷𝑖𝑓𝑓 (s^n^) | 8.98E-04 | 8.12E-02 |
| 𝐷_o_ (cm^2^s^-1^) | 2.47E-12 | 3.06E-12 |

| $Z_{\emptyset}\left( \omega\right)=R_{e}+ \frac{R_{t}}{1+({j\omega)}^{\alpha}QR_{t}}$ | (S1) |
| --- | --- |

Where, *w* is the frequency, *R_e_* and *R_t_* are the Ohmic resistance and charge-transfer resistances, respectively. “*α*” y “*Q*” are the exponential factor and pseudocapacitance CPE (*ϕ*), respectively.

The effective capacitance associated to CPE, for this case the charge-transfer for both layers is calculated as is described in **Equation** **S2**.^[2]^

| $C_{1,2}eff=\left[ Q\left( \frac{1}{R_{e}}+ \frac{1}{Rt} \right)^{(\alpha-1)} \right]^{\frac{1}{\alpha}}$ | (S2) |
| --- | --- |

The diffusion coefficients were obtained by **Equation S3** and **S4**.

| $Y_{0}=\left( \frac{1}{\sqrt{2}*\sigma} \right)$ | (S3) |
| --- | --- |

Where $Y_{0}:$ 𝑄_𝐷𝑖𝑓𝑓 (S*s^n^) and $\sigma:$Warburg coefficient

| $\sigma=\frac{RT}{n^{2}{*F}^{2}*A*\sqrt{2}}*\left( \frac{1}{C_{o}^{*}*\sqrt{D_{0}}}+ \frac{1}{C_{R}^{*}*\sqrt{D_{R}}} \right)$ | (S4) |
| --- | --- |

In which: $D_{0}$ : diffusion coefficient of the oxidant (m²/s); $D_{R}$ : diffusion coefficient of the reductant (m²/s); A : surface area of the electrode (m² ); n : number of electrons transferred; C* : bulk concentration of the diffusing species (mol/cm³).


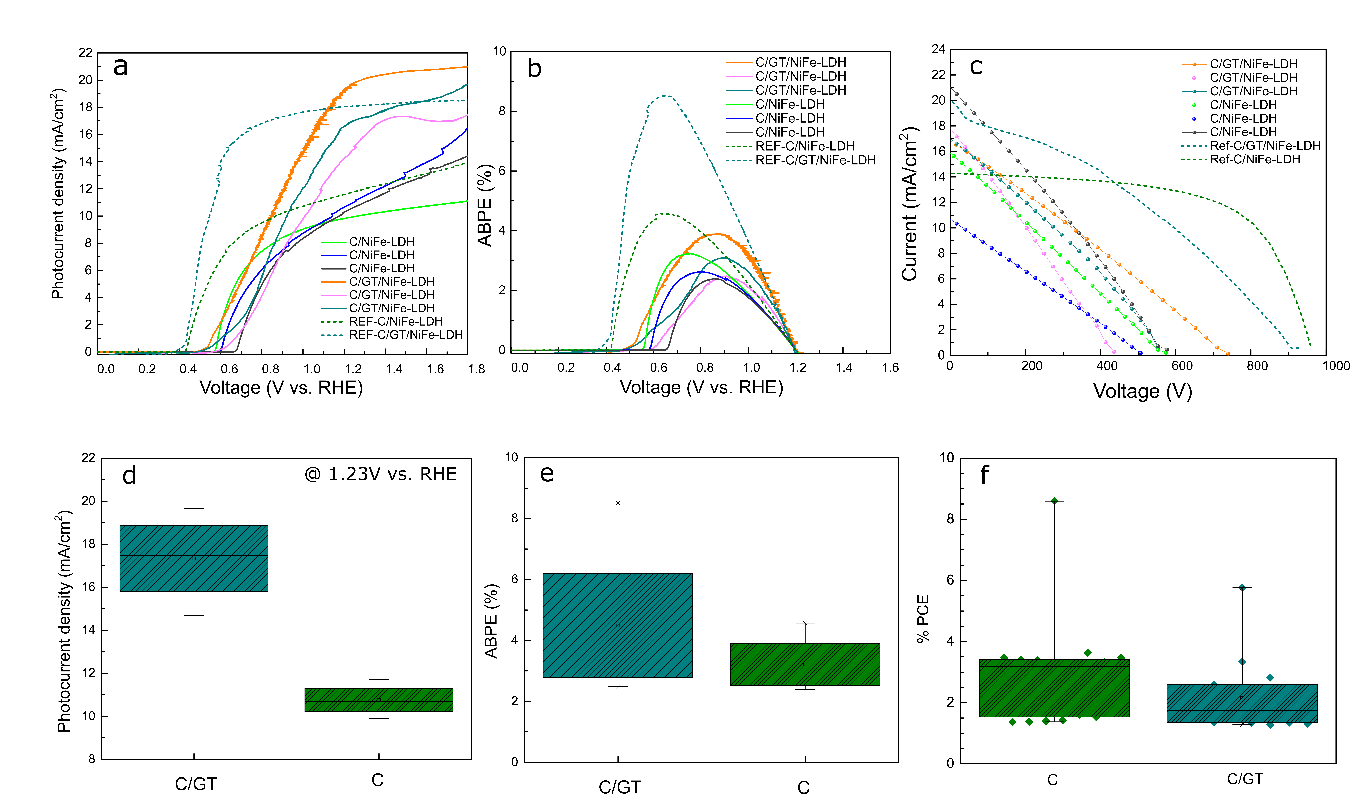


Figure S11. Performance of C/GT/NiFe-LDH and C/GT/NiFe-LDH devices. a) LSV, b) %ABPE, c) JV curve. Statistic of the d) Photocurrent density, e) %AABPE and f) JV curve - large area C-PSC corresponding to 4 replicas of 4 devices (n=16) for each device configuration

**Figure S11a.** presents the photocurrent curves obtained for the C/GT/NiFe-LDH and C/NiFe-LDH configurations, tested under identical conditions. For each system, four independent replicates were evaluated, including the best-performing device in each group, which is highlighted with dashed lines. Based on these measurements, the ABPE was calculated for each device, as shown in **Figure S11b**. where the top-performing devices are also identified with dashed lines. **Figure S11c.** displays the J–V curves of the solar cells used to fabricate the photoelectrochemical devices. These curves show that most devices exhibit suitable current densities for use in photogeneration applications, with average open-circuit voltages (Voc) ranging from 500 to 600 mV, and a reasonable fill factor, confirming good photovoltaic performance at the device level.

From a statistical perspective, the results clearly differentiate both systems: For photocurrent in the **Figure S11d.,** the C/GT/NiFe-LDH system achieved an average of 17.325 mA/cm² with a standard deviation of ±2.097, whereas C/NiFe-LDH showed a lower average of 10.752 mA/cm² and a smaller deviation of ±0.756. This indicates that while the GT-based system exhibits higher variability, it also delivers significantly greater current output. Regarding ABPE efficiency in the **Figure S11e**. a similar trend is observed where the system C/GT/NiFe-LDH present an average and deviation of 4.492% ± 2.738 and the system C/NiFe-LDH an average and deviation of 3.205% ± 0.976. These results suggest that although the C/GT/NiFe-LDH devices show greater spread, they achieve a superior average efficiency, both in charge generation and in conversion performance.

Finally, analysis of the photovoltaic efficiency of the solar cells used in the Figure S11f. confirms that both systems are suitable for driving the oxygen evolution reaction (OER). However, the statistical data highlight that the C/GT/NiFe-LDH configuration not only achieves better overall performance but also maintains acceptable reproducibility, which is essential for practical applications and future scalability.

**References**

[1] D. Hansora, J. W. Yoo, R. Mehrotra, W. J. Byun, D. Lim, Y. K. Kim, E. Noh, H. Lim, J. W. Jang, S. Il Seok, J. S. Lee, *Nat Energy* **2024**, *9*, 272.

[2] A. M. K. Fehr, A. Agrawal, F. Mandani, C. L. Conrad, Q. Jiang, S. Y. Park, O. Alley, B. Li, S. Sidhik, I. Metcalf, C. Botello, J. L. Young, J. Even, J. C. Blancon, T. G. Deutsch, K. Zhu, S. Albrecht, F. M. Toma, M. Wong, A. D. Mohite, *Nat Commun* **2023**, *14*, DOI 10.1038/s41467-023-39290-y.

[3] M. Wang, Y. Li, X. Cui, Q. Zhang, S. Pan, S. Mazumdar, Y. Zhao, X. Zhang, *ACS Appl Energy Mater* **2021**, *4*, 7526.

[4] R. Tao, Z. Sun, F. Li, W. Fang, L. Xu, *ACS Appl Energy Mater* **2019**, *2*, 1969.

[5] H. Chen, M. Zhang, T. Tran-Phu, R. Bo, L. Shi, I. Di Bernardo, J. Bing, J. Pan, S. Singh, J. Lipton-Duffin, T. Wu, R. Amal, S. Huang, A. W. Y. Ho-Baillie, A. Tricoli, *Adv Funct Mater* **2021**, *31*, DOI 10.1002/adfm.202008245.

[6] P. Da, M. Cha, L. Sun, Y. Wu, Z.-S. Wang, G. Zheng, *Nano Lett* **2015**, *15*, 3452.

[7] I. Poli, U. Hintermair, M. Regue, S. Kumar, E. V. Sackville, J. Baker, T. M. Watson, S. Eslava, P. J. Cameron, *Nat Commun* **2019**, *10*, DOI 10.1038/s41467-019-10124-0.

[8] R. Tang, S. Zhou, H. Li, R. Chen, L. Zhang, L. Yin, *Appl Catal B* **2020**, *265*, DOI 10.1016/j.apcatb.2019.118583.

[9] C. Wang, S. Yang, X. Chen, T. Wen, H. G. Yang, *J Mater Chem A Mater* **2017**, *5*, 910.

[10] J. Luo, H. Yang, Z. Liu, F. Li, S. Liu, J. Ma, B. Liu, *Mater Today Chem* **2019**, *12*, 1.

[11] M. Daboczi, J. Cui, F. Temerov, S. Eslava, *Advanced Materials* **2023**, *35*, 2304350.

[12] T. G. Kim, J. H. Lee, G. Hyun, S. Kim, D. H. Chun, S. Lee, G. Bae, H.-S. Oh, S. Jeon, J. H. Park, *ACS Energy Lett* **2022**, *7*, 320.

[13] Z. Zhu, M. Daboczi, M. Chen, Y. Xuan, X. Liu, S. Eslava, *Nat Commun* **2024**, *15*, 2791.

[14] M. Tavakoli Hafshejani, R. Keshavarzi, V. Mirkhani, M. Moghadam, S. Tangestaninejad, I. Mohammadpoor-Baltork, *Int J Hydrogen Energy* **2024**, *59*, 82.

[15] N. Jiang, L. Zhang, Z. Li, Z. Ye, H. He, J. Jiang, L. Zhu, *Chemical Engineering Journal* **2024**, *492*, 152024.

[16] M. Suryawanshi, S. W. Shin, U. Ghorpade, D. Song, C. W. Hong, S.-S. Han, J. Heo, S. H. Kang, J. H. Kim, *J Mater Chem A Mater* **2017**, *5*, 4695.

[17] J. M. Yu, J. Lee, Y. S. Kim, J. Song, J. Oh, S. M. Lee, M. Jeong, Y. Kim, J. H. Kwak, S. Cho, C. Yang, J. W. Jang, *Nat Commun* **2020**, *11*, DOI 10.1038/s41467-020-19329-0.

[18] B. Guo, A. Batool, G. Xie, R. Boddula, L. Tian, S. U. Jan, J. R. Gong, *Nano Lett* **2018**, *18*, 1516.

[19] K. H. Ye, H. Li, D. Huang, S. Xiao, W. Qiu, M. Li, Y. Hu, W. Mai, H. Ji, S. Yang, *Nat Commun* **2019**, *10*, DOI 10.1038/s41467-019-11586-y.

[20] Z. Tian, P. Zhang, P. Qin, D. Sun, S. Zhang, X. Guo, W. Zhao, D. Zhao, F. Huang, *Adv Energy Mater* **2019**, *9*, DOI 10.1002/aenm.201901287.

[21] G. Liu, S. Ye, P. Yan, F. Xiong, P. Fu, Z. Wang, Z. Chen, J. Shi, C. Li, *Energy Environ Sci* **2016**, *9*, 1327.

[22] J. Fu, Z. Fan, M. Nakabayashi, H. Ju, N. Pastukhova, Y. Xiao, C. Feng, N. Shibata, K. Domen, Y. Li, *Nat Commun* **2022**, *13*, DOI 10.1038/s41467-022-28415-4.

[23] A. Mutlu, T. Yeşil, D. Klymaz, C. Zafer, *ACS Omega* **2022**, *7*, 17907.

[24] M. Saliba, T. Matsui, J. Y. Seo, K. Domanski, J. P. Correa-Baena, M. K. Nazeeruddin, S. M. Zakeeruddin, W. Tress, A. Abate, A. Hagfeldt, M. Grätzel, *Energy Environ Sci* **2016**, *9*, 1989.

[25] J. J. Patiño López, M. F. Vasquez-Montoya, C. A. Velásquez, S. Cartagena, J. F. Montoya, M. A. Martinez-Puente, D. Ramírez, F. Jaramillo, *ACS Appl Mater Interfaces* **2023**, *15*, 56547.

[26] Z. Qiu, C.-W. Tai, G. A. Niklasson, T. Edvinsson, *Energy Environ Sci* **2019**, *12*, 572.

[27] D. S. Hall, D. J. Lockwood, C. Bock, B. R. MacDougall, *Proceedings of the Royal Society A: Mathematical, Physical and Engineering Sciences* **2015**, *471*, 20140792.

[28] J. Yan, L. Kong, Y. Ji, J. White, Y. Li, J. Zhang, P. An, S. Liu, S.-T. Lee, T. Ma, *Nat Commun* **2019**, *10*, 2149.

[29] K. R. Kumar, N. D. Sri, V. N. Kale, T. Maiyalagan, *Int J Hydrogen Energy* **2025**, *101*, 837.

[30] X. Huang, K.-H. Kim, H. Jang, X. Luo, J. Yu, Z. Li, Z. Ao, J. Wang, H. Zhang, C. Chen, D. O’Hare, *ACS Appl Mater Interfaces* **2023**, *15*, 53815.

[31] F. Adar, *Spectroscopy* **2022**, *37*, 11.

[32] Y. M. Mao, K. Amreen, R. K. Calay, A. Banerjee, S. Goel, *Sci Rep* **2024**, *14*, 29994.
